# Adult obesity and risk of severe infections: a multicohort study with global burden estimates

**DOI:** 10.1016/S0140-6736(25)02474-2

**Published:** 2026-03-07

**Authors:** Solja T Nyberg, Philipp Frank, Sara Ahmadi-Abhari, Jaana Pentti, Jussi Vahtera, Jenni Ervasti, Sakari B Suominen, Timo E Strandberg, Pyry N Sipilä, Seppo Meri, Naveed Sattar, Mika Kivimäki

**Affiliations:** a Clinicum, Faculty of Medicine, University of Helsinki, Helsinki, Finland; b Finnish Institute of Occupational Health, Helsinki, Finland; c Brain Sciences, University College London, London, UK; d Department of Epidemiology and Biostatistics, School of Public Health, Imperial College London, London, UK; e Department of Public Health and Centre for Population Health Research, University of Turku, Turku, Finland; f Turku University Hospital, Turku, Finland; g The Wellbeing Services County of Southwest Finland, Turku, Finland; h School of Health Science, University of Skövde, Skövde, Sweden; i Department of Medicine, University of Helsinki and Helsinki University Hospital, Helsinki, Finland; j Center for Life Course Health Research, University of Oulu, Oulu, Finland; k Department of Bacteriology and Immunology and Translational Immunology Research Program, University of Helsinki, Helsinki, Finland; l Hospital District of Helsinki and Uusimaa Diagnostic Center, Helsinki University Hospital Laboratory, Helsinki, Finland; m School of Cardiovascular and Metabolic Health, University of Glasgow, Glasgow, UK

## Abstract

**Background:**

Adult obesity has been linked to specific infections, but evidence across the full spectrum of infectious diseases remains scarce. In this multicohort study with impact modelling, we examined the association between this preventable risk factor and the incidence, hospitalisations, and mortality of 925 bacterial, viral, parasitic, and fungal infectious diseases, and estimated their global and regional attributable impact.

**Methods:**

We used pooled data from two Finnish cohort studies and repeated analyses in an independent population from the UK Biobank. BMI was assessed at baseline (1998–2002 in the Finnish studies; 2006–10 in UK Biobank), and participants were categorised as having healthy weight (18·5–24·9 kg/m^2^), overweight (25·0–29·9 kg/m^2^) or obesity, classified as class I (30·0–34·9 kg/m^2^), class II (35·0–39·9 kg/m^2^), or class III (≥40·0 kg/m^2^). Participants were followed up through national hospitalisation and mortality registries for hospital admissions and deaths due to infectious diseases. Using hazard ratios derived from the Finnish cohorts and UK Biobank, along with obesity prevalence estimates from the Global Burden of Diseases, Injuries, and Risk Factors Study database, we estimated the proportion of fatal infections attributable to obesity globally, regionally, and by country for the years 2018 (before), 2021 (during), and 2023 (after the COVID-19 pandemic).

**Findings:**

The analysis included 67 766 adults (mean age 42·1 [SD 10·8] years; 49 516 [73·1%] females, 18 250 [26·9%] males) from the Finnish cohorts and 479 498 adults (mean age 57·0 [SD 8·1] years; 261 084 [54·4%] females, 218 414 [45·6%] males) from UK Biobank. Participants had no recent history of infection-related hospitalisations at baseline. During follow-up, there were 8230 incident infection cases in the Finnish cohorts and 81 945 in UK Biobank. Compared with individuals of healthy weight, those with class III obesity had a three-times higher risk of infection-related hospital admissions (Finnish cohorts 2·75 [95% CI 2·24–3·37], UK Biobank 3·07 [2·95–3·19]), death (Finnish cohorts 3·06 [1·25–7·49], UK Biobank 3·54 [3·15–3·98]), or either outcome (Finnish cohorts 2·69 [2·19–3·30], UK Biobank 3·07 [2·95–3·19]). The corresponding pooled hazard ratio for either fatal or non-fatal severe infection among individuals with any obesity (classes I–III) was 1·7 (1·7–1·8). This association was consistent across different indicators of obesity (BMI, waist circumference, and waist-to-height ratio), demographic and clinical subgroups, and a wide range of infections (non-fatal and fatal, acute and chronic, bacterial and viral [including subtypes], and parasitic and fungal). Applying these risk estimates to global burden of disease data, the population attributable fractions of infection-related deaths due to obesity were estimated at 8·6% (6·6–11·1) in 2018, 15·0% (12·8–17·4) in 2021, and 10·8% (8·6–13·6) in 2023.

**Interpretation:**

Adult obesity is a risk factor for infection-related hospitalisations and mortality across diverse pathogen types, populations, and baseline clinical profiles, with evidence suggesting that approximately one in ten infection-related deaths worldwide might be attributable to obesity.

**Funding:**

Wellcome Trust, Medical Research Council, and Research Council of Finland.

## Introduction

According to the Global Burden of Diseases, Injuries, and Risk Factors Study (GBD) collaboration—the world's largest epidemiological study—no country to date has successfully reversed the rising prevalence of adult overweight and obesity.^1^ With obesity also increasing among children and adolescents,^2^ the number of adults with overweight or obesity is projected to continue rising, contributing substantially to the burden of chronic conditions such as type 2 diabetes, cardiovascular disease, some cancers, and multimorbidity.^3^, ^4^

Studies during the COVID-19 pandemic showed that individuals with obesity had a higher risk of severe SARS-CoV-2 infection, reflected by increased hospitalisations and mortality.^5^ Before the COVID-19 pandemic, obesity was also associated with greater mortality from infectious causes in adolescents and with increased rates of numerous hospital-treated infectious diseases in adults.^6^, ^7^, ^8^ These associations are biologically plausible: anatomical, metabolic, and immunological alterations linked to obesity (including a nutrient-rich environment that favours microbial persistence, insulin resistance, hyperglycaemia that supports pathogen growth, and chronic low-grade inflammation) impair multiple immune pathways.^9^, ^10^ Reduced T-cell and NK-cell function, neutrophil dysfunction, dysregulated complement and adiponectin signalling, and diminished mucus clearance and lymphatic flow further compromise host defence.^9^ Overall, these mechanisms point to a generalised susceptibility to worse infectious disease outcomes among individuals with obesity, an assumption that has not yet been robustly evaluated.

**Research in context**

**Evidence before this study**

Obesity might worsen the clinical course of infections by impairing immune and metabolic functions and altering life circumstances. However, few studies have examined the association between obesity and the full spectrum of severe infectious diseases. We searched PubMed for studies examining the association between obesity and infections, including original research, meta-analyses, and systematic reviews, without language or date restrictions, from database inception to Nov 19, 2025. The search terms were (BMI OR obesity OR obese) AND (infection OR infectious), and reference lists of relevant publications were also screened. Evidence suggests that obesity is associated with an increased risk of several infections, including those affecting the respiratory tract and skin. Obesity has also been linked to more severe courses of infectious diseases such as COVID-19 and is recognised as a risk factor for surgical site infections and hospital-acquired infections. Conversely, infections with specific pathogens, such as adenovirus 36 and *Helicobacter pylori*, have been proposed as potential risk factors for obesity, although the direction of these associations remains uncertain and might be bidirectional. Few meta-analyses or large-scale studies using individual-level data have systematically examined the association between adulthood obesity and the risk of hospitalisation or death from a broad range of infectious diseases, or estimated the global or regional proportion of infection-related deaths attributable to obesity.

**Added value of this study**

This multicohort analysis of more than 540 000 participants, combining data from the Finnish Public Sector study, the Finnish Health and Social Support study, and the UK Biobank, examined the prospective association between adult obesity and the risk of severe infections across multiple infection categories. The study encompassed 925 distinct diagnostic codes for hospitalisations and deaths due to infections, classified by chronicity (acute *vs* chronic) and pathogen type (bacterial, viral, parasitic, or fungal). Bacterial infections were further subdivided by site (invasive or localised), presence or absence of sepsis, cellular tropism (extracellular or intracellular), and pathogen type (Gram-positive, Gram-negative, mycobacterial, or mycoplasma). Viral infections were grouped as acute, herpesvirus (persistent) infections, or other persistent viral infections. With few exceptions (eg, HIV and tuberculosis), there was consistent evidence of a dose–response relationship between obesity classes I–III and higher risk of severe infections compared with healthy weight. The association between obesity and infection risk was observed across subgroups defined by sociodemographic and lifestyle factors, baseline health status, and infection category. When applied to Global Burden of Diseases, Injuries, and Risk Factors Study data, the findings suggest that the proportion of infection-related deaths attributable to adult obesity was 8·6% (95% CI 6·6–11·1) before the COVID-19 pandemic, 15·0% (12·8–17·4) during the pandemic, and 10·8% (8·6–13·6) after the pandemic.

**Implications of all the available evidence**

A large body of research has linked obesity to an increased risk of non-communicable diseases. Our findings suggest that adult obesity is also associated with a higher risk of hospitalisation and death from a broad spectrum of severe infections. This risk follows a clear dose–response pattern across obesity classes 1–3 and is estimated to account for approximately one in ten infection-related deaths worldwide. Given the rising global prevalence of obesity, its contribution to the burden of severe infections is likely to increase further in the coming decades.

To address this evidence gap, we examined associations between adult overweight or obesity and 925 severe infectious diseases, classified by chronicity and pathogen type, including detailed subcategories of bacterial and viral infections (appendix 1 pp 2, 4–26). We further combined these effect estimates with multinational data on obesity prevalence and mortality to estimate the proportion of infection-related deaths attributable to adult obesity worldwide and across regions.

## Methods

### Study population and study design

In this prospective multicohort study, we pooled individual-level data from the Finnish Public Sector (FPS) study (baseline 2000–02) and the Health and Social Support (HeSSup) study (baseline 1998), with replication in an independent population from the UK Biobank (baseline 2006–10). BMI was assessed at baseline, and participants were followed up via national hospitalisation and mortality registries for infection-related hospital admissions and deaths until 2016 in FPS, 2012 in HeSSup, and 2022 in UK Biobank. Participants with a history of severe infections at baseline were excluded. Ethical approval for all studies included in the primary and replication analyses was obtained from local committees on the ethics of human research. Details of the study design and participants are provided in appendix 2 (pp 3–5).

To estimate the population attributable fraction (PAF) of obesity for fatal infections worldwide, we used publicly available summary data from the Institute for Health Metrics and Evaluation.^1^, ^11^ These data include estimates of obesity prevalence and infectious disease mortality across 204 countries and regions before (2018), during (2021), and after (2023) the COVID-19 pandemic.^1^, ^12^

### Measurement of BMI and other indicators of obesity

In FPS and HeSSup, participants' weight and height were self-reported via baseline questionnaires. In UK Biobank, height was measured using a Seca 202 device, weight using the Tanita BC418MA body composition analyser (Tanita, Manchester, UK), and waist circumference with a horizontally positioned tape measure. An additional BMI assessment was conducted 4 years after baseline in FPS and 5 years after baseline in HeSSup.

Participants with missing data on height or weight, with BMI <18·5 kg/m^2^ (underweight), or with implausible BMI values (>70 kg/m^2^) were excluded. Participants were then classified into healthy weight (BMI 18·5–24·9 kg/m^2^), overweight (BMI 25·0–29·9 kg/m^2^), and obesity (BMI ≥30·0 kg/m^2^). Obesity was further divided into class I (BMI 30·0–34·9 kg/m^2^), class II (BMI 35·0–39·9 kg/m^2^), and class III (BMI ≥40·0 kg/m^2^).

Waist circumference was categorised as healthy (<94·0 cm in males and <80·0 cm in females), increased (94·0–101·9 cm in males and 80·0–87·9 cm in females), and high (≥102·0 cm in males and ≥88·0 cm in females), with high waist circumference indicating central obesity.^13^

As an indicator of abdominal fat distribution, we calculated the waist-to-height ratio and categorised it as healthy (<0·50), increased (0·50–0·59), or high (≥0·60), with high waist-to-height ratio broadly corresponding to obesity as defined by BMI.^14^

### Definition of baseline covariates

Demographic, lifestyle, and clinical covariates were measured at baseline and included age, sex, ethnicity, education, adulthood socioeconomic status, physical activity, smoking, alcohol consumption, glucocorticoid use, depression, hypertension, metabolic syndrome, diabetes, cardiometabolic disease (type 1 or type 2 diabetes, coronary heart disease, or stroke), respiratory disease (asthma or chronic obstructive pulmonary disease [COPD]), and cancer (appendix 2 pp 5–9).

### Ascertainment of severe infections during follow-up

The main outcome was the first record of incident non-fatal hospital-treated or fatal infection, identified via national hospitalisation and mortality registries, which capture inpatient admissions but not emergency visits without subsequent admission. Participants with any record of infection in the hospitalisation registry at or before baseline were excluded. Incident severe infection was defined as the first occurrence of either non-fatal hospitalisation with an infection (coded as the primary or secondary diagnosis) or fatal infection (coded as an immediate or underlying cause of death, or a disease-contributing condition), whichever occurred first.

We included a total of 925 distinct diagnoses, classified by chronicity (acute and chronic infections) and pathogen type (bacterial, viral, parasitic, or fungal), with bacterial and viral infections further subcategorised, resulting in 22 disease groups (appendix 1 pp 2, 4–26).^15^ In addition, we examined ten selected infectious diseases or disease groups: acute pharyngitis or acute tonsillitis, influenza, pneumonia (excluding influenza-related pneumonia), lower respiratory tract infections, urinary tract infections, gastrointestinal infections, skin and soft tissue infections, HIV, tuberculosis, and COVID-19 (available only in UK Biobank).

### Global and regional statistics on obesity and fatal infectious diseases

To estimate mortality from infectious diseases attributable to adult obesity (BMI >30·0 kg/m^2^), we obtained global, regional, and national data on obesity prevalence among adults aged 25 years and older and infectious disease mortality from the GBD data portal.^1^, ^12^ Regional and country-specific BMI cutoffs for defining obesity were not used to ensure consistency in estimates over time and across geographies. GBD data on fatal infectious diseases were obtained from the Global Infectious Diseases and Epidemiology Network, the Centre for Research on the Epidemiology of Disasters' International Disaster Database, and WHO databases (appendix 2 pp 10–12).

### Statistical analysis

Follow-up began at the baseline assessment of adiposity and continued until the first recorded infection, death, or the end of follow-up, whichever occurred first. After verifying the proportional hazards assumption using Schoenfeld residuals, we examined associations between BMI category and incident infections using Cox proportional hazards regression models. Due to the smaller number of cases in analyses of specific infection types, we used fixed effects meta-analysis to calculate pooled effect estimates across the Finnish cohorts and UK Biobank.

In the primary model, hazard ratios (HRs) for the associations between obesity and incident severe infections were adjusted for age and sex, and additionally for cohort in the Finnish datasets. To test the robustness of these associations, we further adjusted effect estimates for all baseline covariates, including both potential confounders and mediators.

To examine the generalisability of the findings, baseline subgroup analyses were conducted by ethnicity, sex, age, education, adulthood socioeconomic status, smoking, physical activity, alcohol consumption, glucocorticoid use, depression, hypertension, metabolic syndrome, diabetes, cardiometabolic disease, respiratory disease (asthma or COPD), and cancer, and subgroup differences were tested using heterogeneity test.

The proportion of participants with missing covariate data was relatively small, ranging from 0% to 8% (table); participants with missing data were therefore excluded from the corresponding subgroup analyses. Multivariable-adjusted analyses were repeated after performing multiple imputation for missing covariate data.

**Table:** Baseline characteristics of the participants by cohort

		**Finnish cohorts (n=67 766)**	**UK Biobank (n=479 498)**
Sex			
	Female	49 516 (73·1%)	261 084 (54·4%)
	Male	18 250 (26·9%)	218 414 (45·6%)
	Missing	0	0
Age, years		42·1 (10·8)	57·0 (8·1)
Ethnicity			
	White	..	452 104 (94·3%)
	Asian	..	9167 (1·9%)
	Black	..	7483 (1·6%)
	Other	..	8514 (1·8%)
	Missing	..	2230 (0·5%)
BMI category			
	Healthy weight	39 156 (57·8%)	157 917 (32·9%)
	Overweight	21 216 (31·3%)	205 319 (42·8%)
	Obesity, class I	5779 (8·5%)	83 797 (17·5%)
	Obesity, class II	1272 (1·9%)	23 567 (4·9%)
	Obesity, class III	343 (0·5%)	8898 (1·9%)
	Missing	0	0
Socioeconomic status*			
	Intermediate or high	36 201 (80·9%)	361 439 (75·4%)
	Low	8064 (18·0%)	117 480 (24·5%)
	Missing	478 (1·1%)	579 (0·1%)
Education			
	Intermediate or high	55 230 (81·5%)	390 390 (81·4%)
	Low	12 292 (18·1%)	79 986 (16·7%)
	Missing	244 (0·4%)	9122 (1·9%)
Current smoking			
	No	50 464 (74·5%)	427 772 (89·2%)
	Yes	13 273 (19·6%)	49 358 (10·3%)
	Missing	4029 (5·9%)	2368 (0·5%)
Low physical activity			
	No	53 290 (78·6%)	236 471 (49·3%)
	Yes	13 188 (19·5%)	207 163 (43·2%)
	Missing	1288 (1·9%)	35 864 (7·5%)
Heavy alcohol consumption			
	No	57 260 (84·5%)	296 862 (61·9%)
	Yes	9382 (13·8%)	179 712 (37·5%)
	Missing	1124 (1·7%)	2924 (0·6%)
Use of glucocorticoids			
	No	62 899 (92·8%)	460 666 (96·1%)
	Yes	4867 (7·2%)	18 832 (3·9%)
	Missing	0	0
Hypertension			
	No	60 351 (89·1%)	210 558 (43·9%)
	Yes	7415 (10·9%)	256 280 (53·4%)
	Missing	0	12 660 (2·6%)
Metabolic syndrome			
	No	..	293 749 (61·3%)
	Yes	..	147 543 (30·8%)
	Missing	..	38 206 (8·0%)
Depression			
	No	48 019 (70·9%)	335 067 (69·9%)
	Yes	19 712 (29·1%)	126 056 (26·3%)
	Missing	35 (0·1%)	18 375 (3·8%)
Chronic physical disease			
	No	63 746 (94·1%)	427 470 (89·1%)
	Yes	4020 (5·9%)	52 028 (10·9%)
	Missing	0	0
Diabetes			
	No	66 273 (97·8%)	455 125 (94·9%)
	Yes	1493 (2·2%)	24 373 (5·1%)
	Missing	0	0
Cardiometabolic disease			
	No	65 891 (97·2%)	449 423 (93·7%)
	Yes	1875 (2·8%)	30 075 (6·3%)
	Missing	0	0
Respiratory disease			
	No	66 106 (97·6%)	465 053 (97·0%)
	Yes	1660 (2·5%)	14 445 (3·0%)
	Missing	0	0
Cancer			
	No	67 163 (99·1%)	468 224 (97·6%)
	Yes	603 (0·9%)	11 274 (2·4%)
	Missing	0	0

Data shown are n (%) or mean (SD).

* These data were not available in the Health and Social Support study.

To assess potential non-linearity in the associations of continuous BMI, waist circumference, and waist-to-height ratio with infection risk, we fitted spline regression models, defining the upper limit of the healthy weight range as the reference (HR=1).

To examine the association between changes in BMI category and the risk of severe infections, we divided participants with repeated height and weight measurement into seven categories based on the first two assessments: remained at healthy weight, progressed from healthy weight to overweight or obesity, moved from overweight to healthy weight, remained overweight, progressed from overweight to obesity, moved from obesity to overweight or healthy weight, and remained living with obesity. Participants with infectious diseases at or before the second BMI assessment were excluded, and follow-up was initiated at the time of the second BMI assessment. To assess relative differences in infection risk, we used adjusted Cox proportional hazards regression models, comparing participants who gained or lost weight with those who remained in the same baseline BMI category. We also conducted a sensitivity analysis using the second BMI assessment as a time-dependent covariate and starting infection follow-up from the first BMI assessment.

To estimate the infection burden attributable to adult obesity, assuming the observed associations were causal, we computed the PAF for obesity using pooled HRs from the primary analysis for incident infections with and without COVID-19, as appropriate. We used Monte Carlo simulation with 1000 iterations to estimate 95% CIs for PAFs before, during, and after the COVID-19 pandemic, based on obesity prevalence estimates and infectious disease deaths from 2018, 2021, and 2023, the latest year with GBD data available across all regions and countries.^16^, ^17^

Analyses were conducted using SAS 9.4, R Studio 2025.09.1 Build 401, and Stata 19.5 statistical software. A detailed description of statistical analyses, together with statistical code, is available in appendix 2 (pp 10–12, 31–35).

### Role of the funding source

The funders of the study had no role in study design, data collection, data analysis, data interpretation, or writing of the report.

## Results

The Finnish cohorts (FPS and HeSSup) included a total of 72 047 participants. After excluding individuals with a history of infections before baseline (n=1974), and those with missing BMI data or very low or implausibly high BMI values (<18·5 or >70·0, n=2307), the analytical sample comprised 67 766 participants (appendix 2 p 4). The mean baseline age was 42·1 years (SD 10·8); 49 516 (73·1%) were female and 18 250 (26·9%) were male (table). At baseline, 39 156 (57·8%) participants had a healthy weight, 21 216 (31·3%) were overweight, and 7394 (10·9%) had obesity. Over a mean follow-up of 14·1 years (SD 3·1), 8230 incident infections occurred, with a mean age at first severe infection of 50·7 years (SD 12·7).

In the UK Biobank, 502 133 participants were recruited (appendix 2 p 4). After excluding 17 342 individuals with a history of severe infections prior to baseline, in addition to 5293 with missing, very low, or implausibly high BMI values, the final analytical sample consisted of 479 498 participants. The mean age at baseline was 57·0 (SD 8·1) years. 261 084 (54·4%) were female, 218 414 (45·6%) were male, 32·9% had a healthy weight, 42·8% had overweight, and 24·2% had obesity (table). Over a mean follow-up of 12·6 years (SD 3·2), 81 945 incident severe infections were recorded, with a mean age at onset of 67·0 years (SD 8·6).

**Figure 1:**
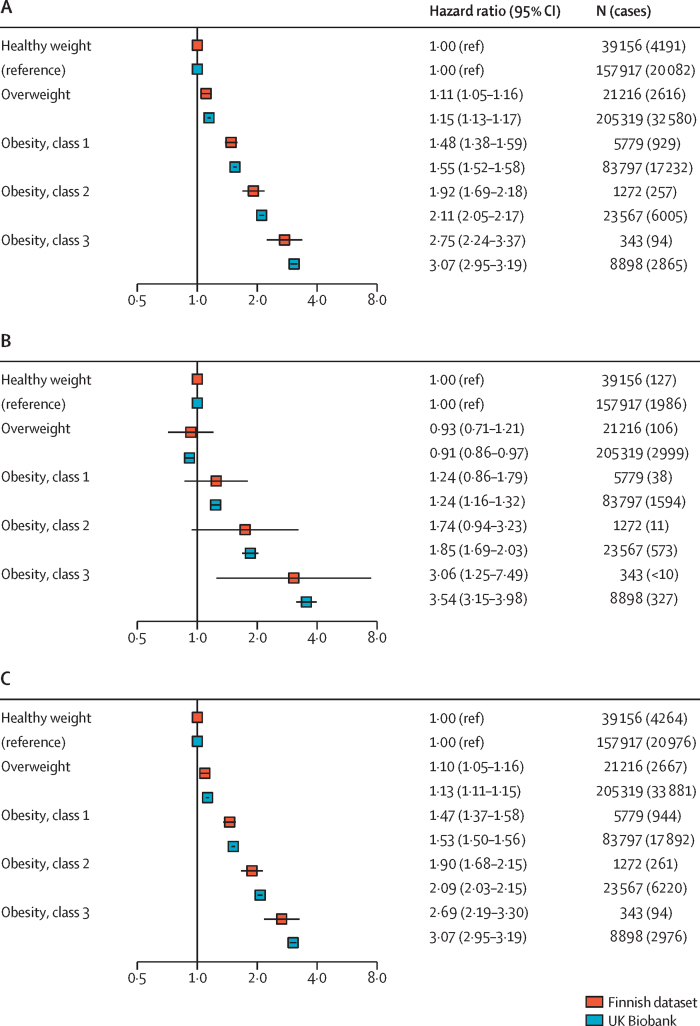
Association between BMI category and risk of severe infectious disease, including non-fatal hospital-treated infections (A), fatal infections (B), and any infections (C) in the Finnish cohorts and UK Biobank

Obesity was associated with incident severe infections in a dose–response manner, with HRs increasing stepwise across BMI categories in all cohorts (figure 1). Compared with individuals with healthy weight, HRs for non-fatal severe infections in people with class III obesity were 2·75 (95% CI 2·24–3·37) in the Finnish cohorts and 3·07 (2·95–3·19) in UK Biobank. HRs for fatal infections were similar: 3·06 (1·25–7·49) in the Finnish cohorts and 3·54 (3·15–3·98) in UK Biobank. We therefore combined non-fatal and fatal severe infections in subsequent analyses, yielding HRs of 2·69 (2·19–3·30) in the Finnish dataset and 3·07 (2·95–3·19) in UK Biobank.

**Figure 2:**
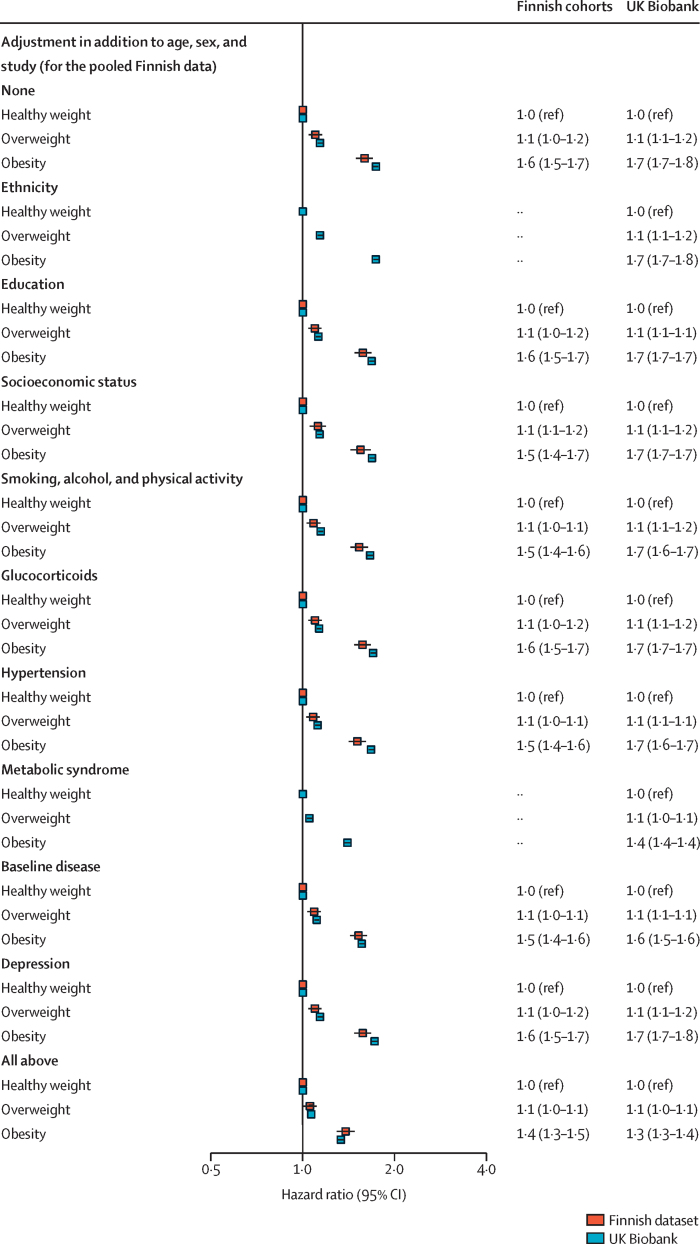
Association between BMI category and risk of severe infectious disease after multivariable adjustments in the Finnish cohorts and UK Biobank

The association of obesity versus healthy weight with severe infections persisted after adjustment for baseline risk factors. Age-adjusted and sex-adjusted HRs were 1·6 (95% CI 1·5–1·7) in the Finnish dataset and 1·7 (1·7–1·8) in the UK Biobank; the corresponding multivariable-adjusted estimates were 1·4 (1·3–1·5) in the Finnish dataset and 1·3 (1·3–1·4) in the UK Biobank (figure 2; appendix 2 pp 14–15). HRs for obesity versus healthy weight were consistent across BMI (1·7 [95% CI 1·7–1·8]), waist circumference (1·7 [1·7–1·8]), and waist-to-height ratio (2·1 [2·0–2·1]), with little difference in the associations when adiposity indicators were modelled as continuous variables (appendix 2 pp 23–24). In addition to the first severe infection, obesity was also associated with an almost 2-times higher risk of recurrent severe infections (appendix 2 p 17).

**Figure 3:**
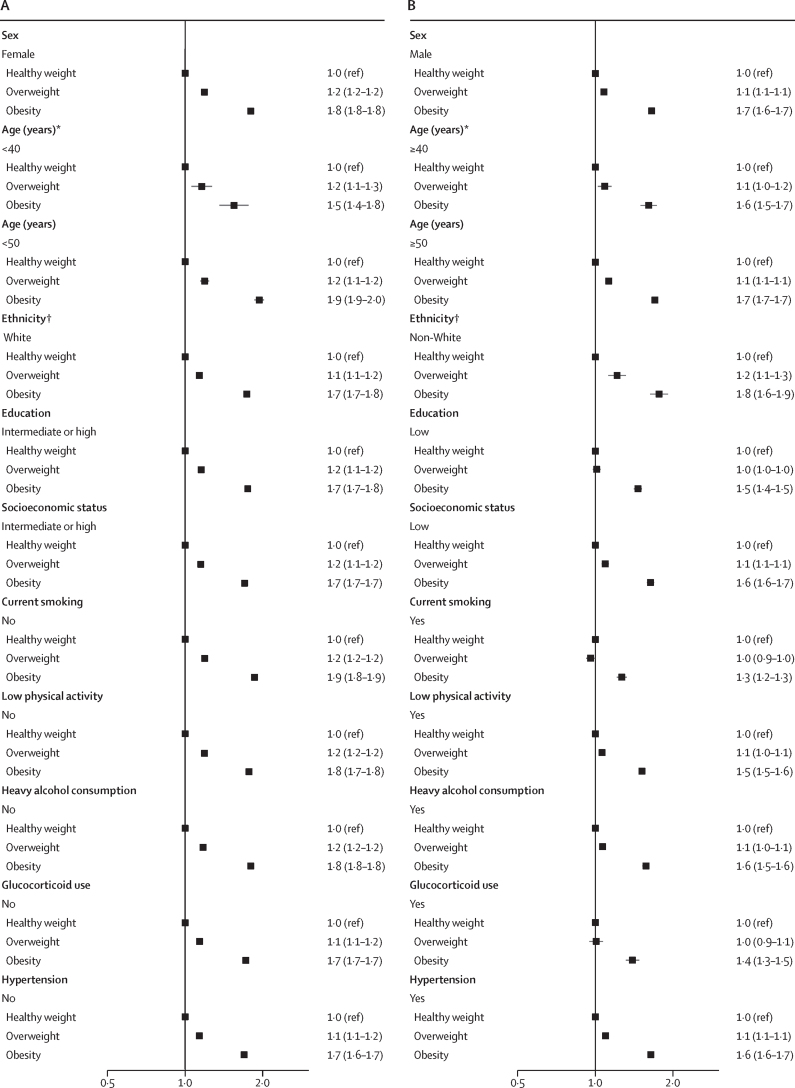

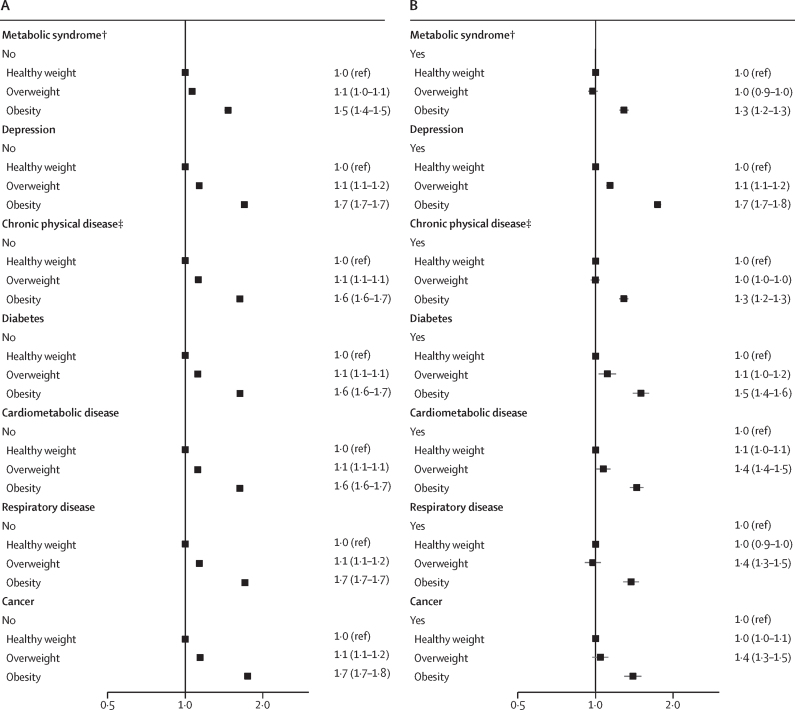
Association between BMI category and risk of severe infections in subgroups, including subgroups with lower infection risk (A) and those with higher infection risk (B) *Only Finnish cohorts. †Only UK Biobank. ‡Diabetes, coronary heart disease, stroke, asthma, chronic obstructive pulmonary disease, or cancer.

In pooled analyses, the association between obesity and incident infections was observed across all subgroups defined by baseline risk factors, although the HRs were slightly lower in subgroups with pre-existing risk factors (figure 3; appendix 2 pp 18–22).

In the Finnish dataset, changes in BMI category were associated with the risk of severe infections (appendix 2 p 17). Compared with persistent obesity, weight loss from obesity to overweight or healthy weight reduced risk (HR 0·8 [95% CI 0·6–1·0]; incidence 111·1 per 10 000 person-years), although not to the level of the persistent healthy-weight group. Weight gain from overweight to obesity increased risk by 1·3 times (1·1–1·5; incidence 109·4 per 10 000 person-years), but not to the level observed in persistent obesity. When compared with persistent healthy weight, the HR was 1·1 (1·0–1·2) for weight gain to overweight or obesity.

**Figure 4:**
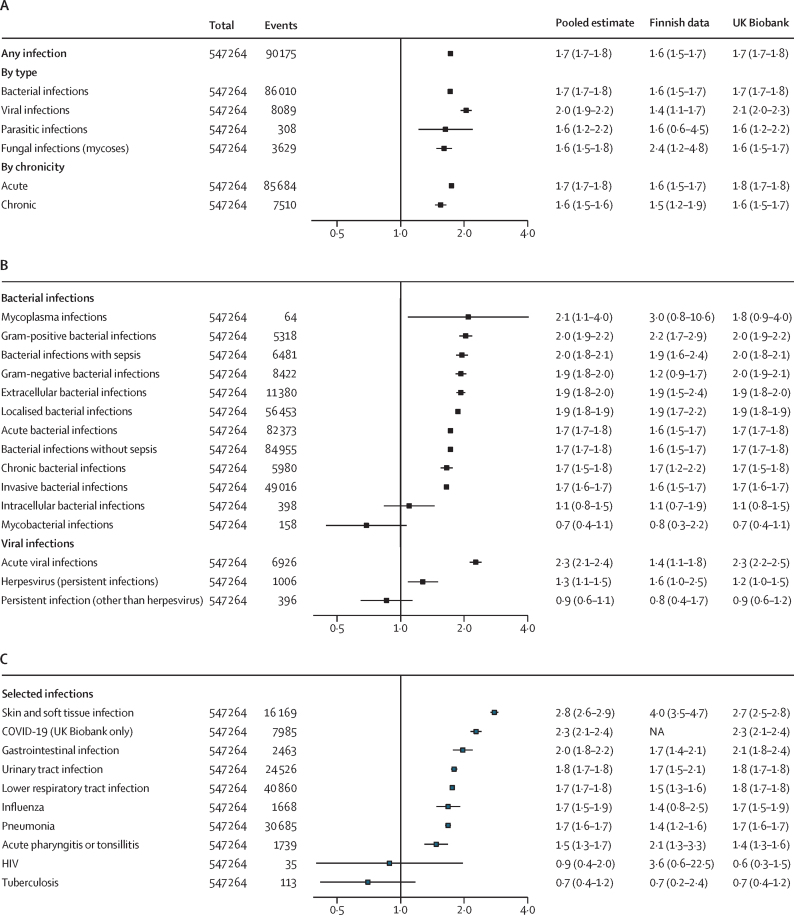
Associations between obesity and severe infection risk by type and chronicity (A), by bacterial and viral subtypes (B), and for specific infections (C) Analyses for each infection type were performed separately. Participant numbers might exceed the total because individuals with multiple infection types contributed to more than one analysis. NA=not applicable.

In analyses of specific infections and broader infection groups, obesity was associated with increased risk for almost all infection types (figure 4; appendix 2 pp 25–29). HRs ranged from 1·6 for parasitic, fungal, and chronic infections to 2·0 for viral infections. For bacterial infections, HRs spanned from 1·7 for invasive infections to 2·1 for mycoplasma infections, with no associations evident for mycobacterial or intracellular bacterial infections. For viral infections, HRs ranged from 1·3 for herpesvirus infections to 2·3 for acute viral infections, whereas persistent viruses (other than herpes) showed no association.

In analyses of ten widely studied infectious diseases, obesity showed the strongest association with skin and soft tissue infections (HR 2·8 [95% CI 2·6–2·9]) and the weakest with acute pharyngitis or tonsillitis (1·5 [1·3–1·7]); for COVID-19, the HR was 2·3 (2·1–2·4; figure 4; appendix 2 p 27). No associations were observed for HIV (which accounted for 8·8% of all persistent non-herpes viral infections; 0·9 [0·4–2·0]) or tuberculosis (comprising 71·5% of all mycobacterial infections; 0·7 [0·4–1·2]).

**Figure 5:**
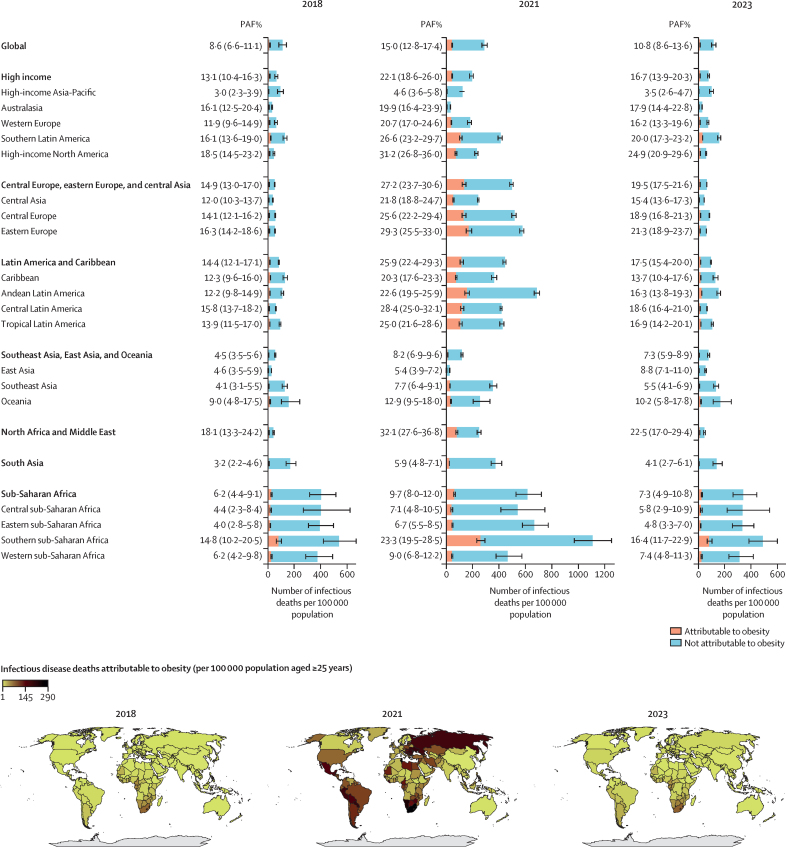
Contribution of obesity to infectious disease burden globally, by region (A) and by country (B) PAF=population attributable fraction.

In GBD-based analyses of absolute case numbers for 2023, 0·6 million (95% CI 0·5–0·7) infectious deaths were attributable to adult obesity, out of 5·4 million (4·6–6·5) total infectious deaths (figure 5; appendix 3 pp 3–4). These numbers were higher during the COVID-19 pandemic in 2021, with 2·0 million (1·8–2·3) obesity-attributable infectious deaths and 13·6 million (12·6–14·8) total infectious deaths; and lower before the pandemic in 2018, with 0·4 million (0·3–0·5) obesity-attributable infectious deaths and 4·9 million (4·2–5·9) total infectious deaths. The corresponding PAFs for obesity in infectious deaths were 8·6% (6·6–11·1) before the COVID-19 pandemic, 15·0% (12·8–17·4) during the pandemic, and 10·8% (8·6–13·6) in the post-pandemic period.

Results by GBD super-region and country are shown (figure 5; appendix 3 pp 6–9). The highest PAFs for obesity-attributable infectious deaths were observed in north Africa and the Middle East super-region—18·1% (95% CI 13·3–24·2) in 2018, 32·1% (27·6–36·8) in 2021, and 22·5% (17·0–29·4) in 2023—whereas the lowest were seen in South Asia, at 3·2% (2·2–4·6) in 2018, 5·9% (4·8–7·1) in 2021, and 4·1% (2·7–6·1) in 2023.

## Discussion

This multicohort study showed robust associations between adult obesity and an increased risk of severe infections across diverse pathogen types, populations, and baseline clinical profiles, independent of sociodemographic, behavioural, and clinical risk factors. Compared with individuals with healthy weight, the risk of any severe infection was 1·7-times higher for individuals with any obesity (BMI ≥30 kg/m^2^), 1·5-times higher for individuals with class I obesity, and 2·0-times higher for individuals with class II obesity. Class III obesity (BMI ≥40 kg/m^2^) was associated with nearly a 3-times higher risk of non-fatal and fatal infections, with consistent findings across all three adiposity measures: BMI, waist circumference, and waist-to-height ratio. In repeat-measure analyses, weight gain from healthy weight or overweight was associated with a modest increase in infection risk, whereas weight loss from obesity was linked to a modest reduction in risk. Elevated risks were observed for most infection types and selected infections (particularly viral, bacterial, and skin and soft tissue infections) and across subgroups, including individuals with and without diabetes or metabolic syndrome, supporting the robustness of our findings. When applied to GBD statistics, these findings suggest that approximately one in ten infection-related deaths worldwide might be attributable to adult obesity, highlighting its substantial contribution to the global communicable disease burden.

Evidence from randomised controlled trials supports a potential causal link. In the SELECT trial,^18^ conducted during the COVID-19 pandemic and enrolling more than 17 000 adults with overweight or obesity and established cardiovascular disease without diabetes, semaglutide 2·4 mg reduced all-cause mortality over 3·3 years, partly due to fewer infection-related deaths. Consistent with these findings, a meta-analysis of 21 trials for semaglutide and other GLP-1 receptor agonists (mean follow-up duration 2·4 years, n=99 599) reported a 10% reduction in infections.^19^

Our findings confirm and extend previous observational evidence linking obesity to greater infection severity.^5^ The strongest evidence to date relates to respiratory infections, including influenza, influenza-related pneumonia, and COVID-19, with meta-analyses consistently reporting higher risks of severe outcomes and mortality in individuals with obesity.^5^, ^20^, ^21^ Obesity has also been associated with complicated skin and soft tissue infections, such as cellulitis.^22^, ^23^, ^24^, ^25^ Evidence for urinary tract infections is mixed, although several studies and meta-analyses indicate higher risks of urinary tract infections and pyelonephritis in both adults and children.^9^, ^24^

The finding that obesity is associated with a wide range of infections suggests that, in addition to pathogen-specific factors, broader biological mechanisms are likely to be involved. Obesity is characterised by immune dysregulation, chronic systemic inflammation, and metabolic disturbances, all of which can impair host defence against diverse pathogens.^9^ These mechanisms are particularly plausible given that our analyses focused on severe, hospital-treated infections, which reflect not only susceptibility to infection acquisition but also progression and prognosis once infection occurs.

Two rare exceptions to the overall pattern of increased infection risk among individuals with obesity were HIV and tuberculosis. Both are plausibly explained by reverse causality: consistent with our findings, inverse associations have been reported for HIV outcomes, probably reflecting the absence of adverse wasting effects in individuals with obesity.^26^, ^27^ Similarly, lower incidence of and mortality from mycobacterial infections, particularly tuberculosis, have been observed, in which weight loss is a cardinal feature of disease progression and underweight impairs immunity.^28^, ^29^, ^30^ These exceptions highlight the context-dependent nature of the relationship between obesity and infection outcomes.

The public health implications of these findings are considerable. By combining observed HRs with global and regional data on obesity prevalence and infectious disease mortality, we estimated that 9% to 11% of infection-related deaths worldwide could potentially be prevented by eliminating obesity (rising to 15% during the COVID-19 pandemic). If results from the GLP-1RA trials are confirmed in the ongoing SURPASS-CVOT trial comparing tirzepatide with dulaglutide in individuals with type 2 diabetes and cardiovascular disease, this would further strengthen the evidence for causality, suggesting that obesity prevention and weight reduction achieved through widely used pharmacotherapies could lower infection-related mortality.

Several limitations should be noted. Our study relied on observational data, so we are unable to confirm the causality of the associations observed. Although findings were replicated in two independent datasets, neither cohort is population-representative, limiting the generalisability of absolute risks; however, exposure–outcome associations typically remain robust. Our main analyses relied on BMI, which does not fully capture adiposity, fat distribution, or metabolic dysfunction. Height, weight, and several covariates were self-reported in the Finnish cohorts and therefore were subject to recall bias, but results were consistent with those from the UK Biobank, in which BMI was clinically assessed. Underweight could not be examined because of small numbers, despite its known association with severe infection risk.^8^ Ascertainment of severe infections and comorbidities from electronic health records might have missed some cases, although major bias is unlikely if under-ascertainment was unrelated to obesity. Lastly, estimates of global impact should be interpreted cautiously because data on infection-related deaths and obesity prevalence in the GBD data might be inaccurate, particularly for low-resource countries. Furthermore, although we observed little heterogeneity in HRs by sex, age, or ethnic group, the assumption of constant relative risks across demographic groups and regions when estimating the global burden remains a limitation.

In conclusion, obesity should receive greater attention in public health strategies aimed at preventing severe infections. Effective prevention of adiposity, implementation of evidence-based weight-loss interventions, and stronger integration of obesity considerations into vaccination programmes for high-risk groups could help reduce the burden of severe infections and related mortality.

### Contributors

### Data sharing

Anonymised questionnaire data can be requested from the principal investigator by contacting Dr Jenni Ervasti (jenni.ervasti@ttl.fi) for Finnish Public Sector (FPS) data and Dr Sakari Suominen (sakari.suominen@utu.fi) for Health and Social Support (HeSSuP) data. Linked health records for FPS and HeSSuP require separate permission from Findata, the Health and Social Data Permit Authority. Researchers registered with UK Biobank can apply for access to the database by completing an application, which must include a summary of the research plan, data fields required, any new data or variables that will be generated, and payment to cover the incremental costs of servicing an application (https://www.ukbiobank.ac.uk/enable-your-research/apply-for-access).

## Declaration of interests

TES has consulted for MSD, GlaxoSmithKline, and Pfizer. NS has consulted for or received speaker honoraria from Abbott Laboratories, AbbVie, Amgen, AstraZeneca, Boehringer Ingelheim, Carmot Therapeutics, Eli Lilly, GlaxoSmithKline, Hanmi Pharmaceuticals, Menarini-Ricerche, Metsera, Novartis, Novo Nordisk, Pfizer, and Roche; and received grant support paid to his university from AstraZeneca, Boehringer Ingelheim, Novartis, and Roche outside the submitted work. All other authors declare no competing interests.
