# Supplementary material for: Adult obesity and risk of severe infections: a multicohort study with global burden estimates
Source: Lancet. 2026 Mar 7;407(10532):951–62. doi: 10.1016/S0140-6736(25)02474-2 (PMC12979006; doi:10.1016/S0140-6736(25)02474-2)
Supplement: Supplementary appendix 2 [file mmc2.pdf]

THE LANCET

Supplementary appendix 2

This appendix formed part of the original submission and has been peer reviewed. We post it as supplied by the authors.

Supplement to: Nyberg ST, Frank P, Ahmadi-Abhari S, et al. Adult obesity and risk of severe infections: a multicohort study with global burden estimates. *Lancet* 2026; published online Feb 9. https://doi.org/10.1016/S0140-6736(25)02474-2.

Supplementary appendix 2

List of contents **Page**

1 Extended Methods....3
1.1 Study populations....3
Figure S1. Flow chart of sample selection....4
Table S1. Comparison between included and excluded participants....5
1.2 Measurement of adult obesity and baseline covariates in the Finnish dataset....5
1.3 Measurement of adult obesity and baseline covariates in the UK Biobank....7
1.4 Ascertainment of severe infections at follow-up across cohorts....9
1.5 Global and regional statistics on adult obesity and fatal infectious diseases....10
1.6 Extended statistical analysis....10
2 Supplementary Results....13
Table S2. Mean age at the assessment of BMI and at incident infection....13
Figure S2. Association between BMI category and risk of severe infectious disease with different adjustments in the Finnish cohorts and UK Biobank....15
Table S3. Association between obesity and risk of severe infection based on Fine and Gray regression in Finnish cohorts and UK Biobank....16
Table S4. Association between obesity and risk of severe infection in Finnish cohorts using repeat BMI assessments....16
Table S5. Association between obesity and risk of recurrent severe infections....17
Figure S3. Association of change in BMI category with risk of incident severe infection in the Finnish cohorts....17
Table S6. Subgroup differences in the association between obesity vs healthy weight and risk of severe infections by baseline covariates....18
Table S7. Subgroup differences in the association between overweight vs healthy weight and risk of severe infections by baseline covariates....19
Figure S4. Association between BMI category and risk of severe infections in subgroups....20
Table S8. Association between BMI category and risk of severe infectious disease by use of glucocorticoids medication....22
Table S9. Association between adiposity category and risk of severe infectious disease stratified by method of assessment in UK Biobank....23
Figure S5. Associations between continuous adiposity indicators and risk of severe infectious disease....24
Figure S6. Association between obesity and risk of severe infections by type, chronicity and cohort....25
Figure S7. Association between obesity and risk of severe bacterial and viral infections by subtype and cohort....26
Figure S8. Association between obesity and risk of selected severe infections by cohort....27

Table S9. Association between obesity and risk of severe infectious disease by type and for selected infections in the Finnish cohorts and UK Biobank 28
Table S10. Comparison of the basic model and multivariable-adjusted model of obesity and risk of severe infectious disease 29
3 Global and Regional Impact of Obesity on Infectious Deaths 30
4 Statistical Syntax 31
5 References 36
6 STROBE checklist 39

1 Extended Methods

Study populations comprised two Finnish cohort studies with similar data collection protocols and use of the same national health registries, and an independent cohort, the UK Biobank. The legal basis for all three studies was the European and UK General Data Protection Regulation (GDPR), using the lawful bases of "legitimate interests" and "consent" for data processing.

Ethical approval was obtained from the ethical committee of the Helsinki and Uusimaa hospital district (HUS/1210/2016) for the Finnish Public sector study, the Turku University Central Hospital Ethics Committee and the Finnish Population Register Centre (VRK2605/410/14) for the Health and Social Support study, and the National Health Service National Research Ethics Service (11/NW/0382) for the UK Biobank.

1.1 Study populations

Finnish Public Sector study (FPS), Finland.[1] The Finnish Public Sector study is a prospective cohort study comprising the entire public sector personnel of 10 towns (municipalities) and 21 hospitals in the same geographical areas. Participants, who were recruited from employers' records in 2000-2002, were individuals who had been employed in the study organisations for at least six months prior to data collection. 48 592 individuals (9 337 men and 39 255 women aged 17 to 65) responded to the questionnaire.

Health and Social Support (HeSSup), Finland.[2] The Health and Social Support (HeSSup) study is a prospective cohort study of a stratified random sample of the Finnish population in the following four age groups: 20–24, 30–34, 40–44, and 50–54. The participants were identified from the Finnish population register and posted an invitation to participate, along with a baseline questionnaire, in 1998. 25 898 individuals responded to the questionnaire in 1998. The Turku University Central Hospital Ethics Committee approved the study.

The UK Biobank is an ongoing prospective cohort study.[3] Approximately 9.2 million invitations were mailed and during 2006-2010, over half a million men and women aged 40-69 years from the United Kingdom participated. The baseline data collection involved questionnaire and physical measurements. The present study was conducted using the UK Biobank Resource under Application Number 60565.

Sample selection in the Finnish dataset and UK Biobank is shown in **figure S1**.

Figure S1. Flow chart of sample selection

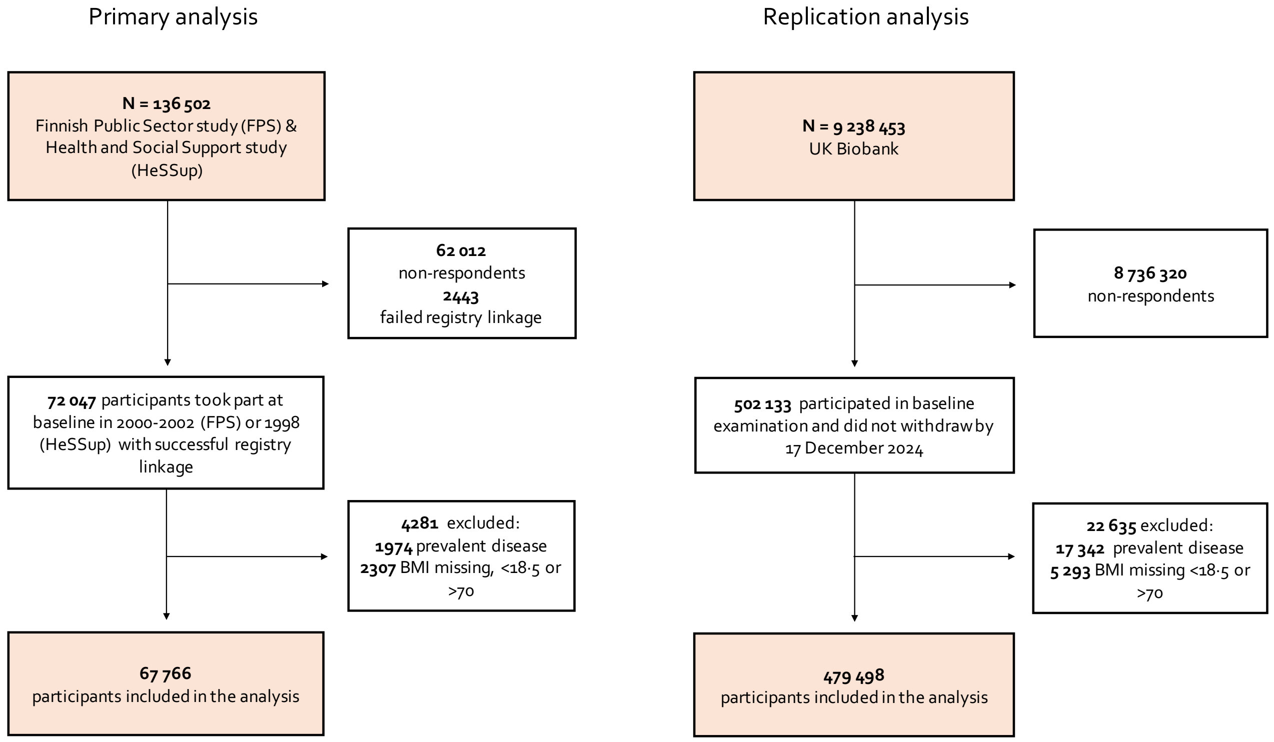

Selection bias. Comparison of the included study sample with the population before exclusions due to missing height or weight data or underweight showed no, or only negligible, differences between the groups (**table S1**). This suggests a low risk of selection bias, although excluded participants with underweight were more often women and those excluded because of missing anthropometric data had a higher incidence of severe infections during follow-up.

Table S1. Comparison between included and excluded participants

		Excluded		
	Study sample %	Underweight %	Missing BMI %	Total population %
**Finnish dataset**				
**Participants, N**	67766	1106	1207	70079
**Sex, %**				
Men	26.9	9.3	13.9	26.4
Women	73.1	90.7	86.1	73.6
**Age, years**				
Mean (SD)	42.2 (10.8)	34.0 (11.2)	44.5 (9.7)	42.1 (10.8)
**Education, %**				
Intermediate or high	81.8	77.3	82.9	81.7
Low	18.2	22.7	17.1	18.3
**Incident infection at follow-up, %**				
No	87.9	87.1	86.6	87.8
Yes	12.1	12.9	13.4	12.2
**UK Biobank**				
**Participants, N**	479,498	2484	2809	484,791
**Sex, %**				
Men	45.6	21.1	52.5	45.6
Women	54.4	79.9	47.5	54.4
**Age,years**				
Mean (SD)	57.0 (8.1)	57.0 (8.1)	56.9 (8.3)	57.0 (8.1)
**Education, %**				
Intermediate or high	81.4	85.8	73.1	81.3
Low	16.7	14.2	26.9	16.7
**Incident infection at follow-up, %**				
No	82.8	80.1	67.7	82.8
Yes	17.2	19.9	32.3	17.2

1.2 Measurement of adult obesity and baseline covariates in the Finnish dataset

Baseline measurements were conducted between March 1, 2000, and June 30, 2002 in FPS, and between June 7, 1998, and May 23, 2000 in HeSSup. In FPS, height and weight were reassessed three times during follow-up: March 1, 2004–June 30, 2005; March 1, 2008–November 30, 2009; and December 1, 2011–November 30, 2013. In HeSSup, height and weight were reassessed once, between January 7, 2003, and August 12, 2003.

Participants' height and weight were self-reported. We calculated **BMI** as weight in kg divided by height in m^2. Participants with missing values for height or weight or BMI values less than healthy weight (ie BMI 18.5 kg/m2) or more than 70 kg/m2 were excluded. We classified participants according to BMI-values into healthy weight (BMI 18.5-24.9kg/m2), overweight (BMI 25.0-29.9kg/m2), and obesity (BMI ≥30 kg/m2). Obesity was further divided into three categories: class I (BMI 30–34.9 kg/m2), class II ((BMI 35–39.9 kg/m2) and class III (BMI ≥40 kg/m2).

Covariates included demographic, lifestyle, and clinical factors that might confound or mediate the associations between adult obesity and severe infections. **Age** and **sex** were obtained from population registry in FPS and from questionnaire in HeSSuP.[4]

Education was registry-based in FPS and self-reported in HeSSuP. Education was categorised into low (primary or lower secondary), intermediate (higher secondary), and high (tertiary qualification, college, or university) levels.[5]

Socioeconomic status (SES) was based on occupational title obtained from employers' register and categorised into low, intermediate, or high.[4] Data on adulthood SES was not available for HeSSup.

Physical activity was classified as either recommended (meeting the Word Health Organization recommendation of ≥2.5 hours of moderate activity per week or ≥1.25 hours of vigorous activity per week),[6] low (no or very little moderate/vigorous physical activity), or intermediate (between the above categories).[7-9] It was assessed in both studies by inquiring about the weekly duration of activities at varying levels of intensity, such as walking, brisk walking, jogging, or running, whether during leisure time or commuting. The response options provided were: 'not at all,' 'less than half an hour,' 'approximately one hour,' '2–3 hours,' and '4 hours or more. Low physical activity was defined as less than 0.5 hour of each (brisk walking, jogging or running) per week. The weekly amount of both moderate and vigorous activities was calculated for classifying intermediate or recommended activity.

Smoking was based on self-report and categorised as current smoking, never smoking and ex-smoking in both studies.[10]

Information on **alcohol consumption** was extracted from questionnaires completed by participants. Alcohol consumption was based on the total number of units (10 g of ethanol) a participant consumed in a week and categorised according to the UK Chief Medical Officers' guidelines in which heavy drinking was denoted as a weekly consumption exceeding 14 units for men and women[11]; moderate drinking was defined as consuming 1–14 units per week. Non-drinkers were divided into self-reported lifelong abstainers and former drinkers.[12] The estimates for weekly consumption of alcohol were based on the reported amounts of beer, wine or other mild alcoholic beverages and hard liquors. For each category, seven pre-defined answer alternatives were given, and weekly consumption was estimated based on the responses.

History with depression was based on a questionnaire item enquiring whether a doctor had ever diagnosed the participant with depression.

In FPS, **current depression** was measured by the self-administered 12-item General Health Questionnaire (GHQ-12)[13]. GHQ-12 consists of 12 questionnaire items inquiring about specific symptoms. Respondents rate the extent to which they are affected by each of the symptoms (1 = not at all, 2 = as much as usual, 3 = slightly more than usual, 4 = much more than usual). In accordance with previous studies, participants with a rating of 3 or 4 in at least four items of the total measure were coded as cases of common mental disorder.[14,15]

In HeSSup, the Beck Depression Inventory (BDI) was administered to all participants. It consists of 21 items on a four-point scale, individual responses ranging from 0 to 3. Total score ranges from 0 to 63. In accordance with previous studies, a score of at least ten points was used to

separate participants with subclinical mild to severe depression from those without depression.[16,17]

Hypertension was based on self-reported doctor diagnosed hypertension, self-reported use of antihypertensive drugs (HeSSup only), or appearance in the nationwide drug reimbursement register as eligible for hypertensive medication.

Use of glucocorticoids at or before baseline was ascertained through linkage to electronic health records from the Finnish Social Security Institute's medication purchases registry. This registry provides dates and Anatomical Therapeutic Chemical (ATC) codes for filled prescriptions; glucocorticoids were identified using ATC codes H02AB and R03BA**.**

History with major chronic diseases included a record of type 1 or type 2 diabetes, coronary heart disease, stroke, cancer, asthma, and COPD before or at the baseline. These diseases were selected for their high prevalence and public health significance in high-income countries, prioritisation by WHO for global disease prevention,[18] and their common use in studies of disease-free life years.[7,12,19] The records were collected from national registers of hospitalisations or prescription reimbursements as follows:

Prevalent **cardiometabolic disease** was defined as type 1 or type 2 diabetes, coronary heart disease or stroke. **Diabetes** was defined as the first record of type 2 diabetes diagnosis corresponding to ICD-10 code E11 or type 1 diabetes: E10 (ICD-10) or 250 (ICD-9 and ICD-8). We collected records from hospital admissions and discharge registers with a mention of diabetes in any of the diagnosis codes. Additionally, participants were also defined as a type 2 diabetes case the first time they appeared in the nationwide drug reimbursement register as eligible for type 2 diabetes medication.[20,21] Coronary heart disease events were identified from hospital discharge register. We included all non-fatal myocardial infarctions that were recorded as I21–I22 (ICD-10) or 410 (ICD-9). Stroke was defined with hospital records (I60, I61, I63, I64 in ICD-10; 430, 431, 433, 434, 436 in ICD-9).[4,22]

Cancers, C00–C97 (ICD-10 any cancer), were identified via national cancer or hospital records.[23]

Respiratory disease was defined as severe asthma (J45 or J46 in ICD-10 or 493 in ICD-9) or COPD exacerbations (J41, J42, J43, and J44 in ICD-10, or 491, 492, and 496 in ICD-9) and ascertained from hospital discharge register.[24,25]

1.3 Measurement of adult obesity and baseline covariates in the UK Biobank

Baseline measurements were conducted between March 13, 2006, and October 1, 2010 in UK Biobank.

Height and **weight** of the participants were measured, and **BMI** was defined similarly as in the Finnish datasets. Height was measured using a Seca 202 device and weight using the Tanita BC418MA body composition analyser (Tanita, Inc. Manchester, UK).

In addition, **waist circumference** was measured using a horizontally positioned tape measure. In a sensitivity analysis, waist circumference was categorised as high (>102cm in men and >88cm in women), increased (94-101.9cm in men and 80-87.9cm in women) and healthy waist (<94cm in men and <80cm in women).[26] The categories of waist-to-height ratio were as

high (0·6 or more), increased (0·5 to 0·59) and healthy (<0·5) central adiposity.[27] The combined measure included three categories based on the abovementioned measures such that obesity was defined as present, if all three measures yielded obesity, and healthy weight, if all three measures indicated healthy weight. Those not fulfilling either condition, were included in the intermediate category.

Sex and **age** of the participants were obtained from registries. This information could be amended by the participant upon arrival at the Assessment Centre. Self-reported **ethnicity** was categorised as White or Non-White, with the latter category subdivided into Asian (or Asian British), Black (or Black British), and Other (White and Black Caribbean, White and Black African, White and Asian, Any other mixed background, Chinese or Other ethnic group).

Education was based on self-report and **SES** was obtained be linking participant's residential address to the corresponding Townsend deprivation index.[28]

Smoking was based on self-report and categorised as current smoking, never smoking and ex-smoking. Information on **alcohol use** was extracted from questionnaires completed by participants and included the frequency and amount of drinking red wine, champagne / white wine, beer / cider, spirits, fortified wine and other (such as alcopops). Weekly alcohol consumption was derived from the responses and categorised similarly as for the main analysis. **History with depression** was based on predefined derived fields of lifetime major depression (MD) status defined from the touchscreen questionnaire at baseline and included "single episode of probable MD", "probable recurrent MD (moderate)", and "probable recurrent MD (severe)" as history with depression.[29,30] **Depression caseness** was defined present, if the response to baseline question "Frequency of depressed mood in last 2 weeks" was several days or more (vs. not at all).[31] **Hypertension** was based on self-reported blood pressure medication, measured diastolic blood pressure 90 or higher or measured systolic blood pressure 140 or higher.

Metabolic syndrome was assessed as previously described,[32] with caseness defined as the presence of at least three of the following five components: (1) abdominal obesity (elevated waist circumference: ≥102 cm in males and ≥88 cm in females); (2) elevated triglycerides (≥150 mg/dL or 1.7 mmol/L); (3) elevated blood pressure (≥130 mmHg systolic blood pressure and/or ≥85 mmHg diastolic blood pressure) or antihypertensive medication use; (4) elevated fasting blood glucose (≥100 mg/dL or ≥5.6 mmol/L) or drug treatment for elevated blood glucose; and (5) reduced HDL-cholesterol (<40 mg/dL or 1.0 mmol/L in males; <50 mg/dL or 1.3 mmol/L in females) or lipid-modifying medications. Following the recommendations of the American Diabetes Association,[33] data on circulating glucose levels were obtained using glycated haemoglobin (HbA1c) as a proxy measure of glucose.

Glucocorticoid use, including systemic and inhaled glucocorticoids, was based on medications reported by the participants.[32]

Study participants were linked to records of **major non-communicable diseases** (cardiometabolic disease, diabetes, cancers, respiratory disease) in the UK National Health Service's Hospital Episode Statistics (HES) database for hospital admissions and the NHS Central Registry for mortality from 18 March, 1995 to baseline, defined with a similar manner as in the Finnish dataset.

1.4 Ascertainment of severe infections at follow-up across cohorts

The outcome was the first record of an incident non-fatal hospital-treated or fatal infection, identified through national hospitalisation and mortality registries. In FPS, participants were linked to the national hospital discharge registry (Finnish Institute for Health and Welfare) and the national mortality registry (Statistics Finland) until December 31, 2016; in HeSSup, linkage continued until December 31, 2012. UK Biobank participants were linked to the NHS Hospital Episode Statistics database for hospital admissions and the NHS Central Registry for mortality until December 19, 2022. These data included hospital inpatient records but excluded emergency department visits that did not result in hospitalisation.

The list of the International Classification of Diseases diagnostic codes (CD-10) diagnostic codes for infectious diseases is provided in **appendix 1**. Participants with any record of infection in the hospitalisation registry at or before baseline were excluded. Infections during follow-up were classified as either fatal or non-fatal. For analyses of non-fatal infections, infections were defined as fatal if the individual died within one month following hospitalization. As a result, these individuals were censored in the relevant analyses. Incident severe infection was defined as the first occurrence of either non-fatal hospitalisation with an infection (coded as the primary or secondary diagnosis for admission) or fatal infection (coded as an immediate or underlying cause of death or a disease contributing to death), whichever occurred first.

As in previous studies, we included a total of 925 distinct diagnoses, classified these into 22 disease groups and 10 specific common infectious diseases, described in detail in **appendix 1, tables S1–S24**).[34,35] Briefly, to examine whether the associations between obesity and incident infections were driven by specific diseases, we classified infections by chronicity (acute and chronic infections) and pathogen type (bacterial, viral, parasitic, or fungal infections) (**appendix 1, figure S1**).[34] Bacterial infections were further categorised as invasive or localised; with or without sepsis; caused by extracellular or intracellular pathogens; and as Gram-positive, Gram-negative, mycobacterial, or mycoplasma infections. Viral infections were grouped into acute viral infections, herpesvirus (persistent) infections, and other persistent viral infections.

We defined chronic infections as those in which the pathogen often or consistently persists in the body, either actively or latently. These included, for example, tuberculosis and other mycobacterial infections, herpesviral infections, mononucleosis, genital papillomavirus infections, dental caries, infections explicitly classified as chronic (eg, chronic periodontitis or chronic meningococcaemia), and several rare infections. All other infections were classified as acute. **Appendix 1 tables S22 and S23** provide a full list of the acute and chronic infections.

In addition, we examined associations between obesity and 10 selected infectious diseases or disease groups, including acute pharyngitis or acute tonsillitis; influenza; pneumonia (excluding pneumonia from influenza); lower respiratory tract infections; urinary tract infections; gastrointestinal infections; skin and soft tissue infections; HIV; tuberculosis; and COVID-19 (only available for UK Biobank) (**appendix 1,table S24**).

1.5 Global and regional statistics on adult obesity and fatal infectious diseases

To estimate mortality from infectious diseases attributable to adult obesity, we obtained global, regional, and national data on obesity prevalence and infectious disease mortality for adults aged 25 years or older from the Global Burden of Diseases, Injuries, and Risk Factors Study (GBD) data portal, maintained by the Institute for Health Metrics and Evaluation.[36,37] Obesity, defined using BMI, was based on both self-reported and measured data with a BMI of 30·0 or higher. Regional and country-specific BMI cutoffs for defining obesity were not used to ensure consistency in estimates over time and across geographies. GBD data on fatal infectious diseases in GBD were obtained from the GIDEON – Global Infectious Diseases and Epidemiology Network /GIDEON), Centre for Research on the Epidemiology of Disasters' International Disaster Database (EM-DAT), and the Word Health Organization databases.

To cover time periods before, during and after the COVID-19 pandemic, we used GBD data for years 2018, 2021, and 2023 (the latest available dataset). Data Sources Tool was available from https://ghdx.healthdata.org/record/ihme-data/gbd-2021-adult-obesity-overweight-prevalence-1990-2050 (accessed on November 18, 2025).

1.6 Extended statistical analysis

Analysis of Finnish dataset and UK Biobank: Follow-up began at the baseline assessment of adiposity and continued until the first recorded infection, death, or the end of follow-up, whichever occurred first. After verifying no heterogeneity in the estimates for the association between BMI category and risk of severe infections, data from the two Finnish cohort studies were pooled and estimates were adjusted for cohort in all analyses. After verifying the proportional hazards assumption using Schoenfeld residuals, we examined the associations between BMI category and incident infections with Cox proportional hazards regression models.

Hazard ratios (HRs) for the associations between obesity and incident severe infections were adjusted for age and sex, and additionally for cohort in the Finnish datasets (the basic model). To test the robustness of these associations, we further adjusted effect estimates for baseline covariates, including both potential confounders and mediators (the multivariable adjusted model). These included the following dichotomous covariates: ethnicity, education, SES, smoking, physical activity, alcohol consumption, use of glucocorticoid medication, metabolic syndrome, hypertension, depression, and baseline chronic disease (diabetes, coronary heart disease, stroke, cancer, asthma or COPD).

To examine bias due to missing data, we repeated multivariable adjusted analyses after imputing missing covariate data using multiple imputation by chained equations (20 imputations) and assuming data were missing at random conditional on observed data. Estimates were pooled across imputations using Rubin's rules.[38]

Because baseline covariates may act as both confounders and mediators of the obesity–infection association, and because multivariable adjustment had little effect on the estimates, subsequent analyses were adjusted for age and sex only to maximise sample size.

To address potential survival bias, we conducted a Fine and Gray competing risk analysis, with severe infections and death as outcomes.[39]

To examine the association between changes in BMI category and risk of severe infections, we divided participant with repeated height and weight measurement into seven categories: (1) remained at healthy weight, (2) progressed from healthy weight to overweight or obesity, (3) moved from overweight to healthy weight, (4) remained overweight, (5) progressed from overweight to obesity, (6) moved from obesity to overweight or healthy weight, and (7) remained living with obesity. Participants with infectious disease at or before the second BMI assessment were excluded and the follow-up was initiated at the time of the second BMI assessment. To assess relative differences in infection risk, we used adjusted Cox proportional hazards regression models, comparing participants who gained or lost weight with those who remained in the same baseline BMI category. In addition, we conducted a sensitivity analysis using the repeated BMI assessments as time-dependent covariates and starting infection follow-up from the first BMI assessment.

To examine whether adult obesity was associated with risk of recurrent severe infections, we used Cox proportional hazards regression models with the second severe infection as the outcome.

To examine the generalizability of the findings, subgroup analyses were conducted by sex (men vs women), age (<40 vs 40+ years and <50 vs 50+ years), ethnicity (White vs non-White; White vs Asian vs Black vs Other), education (low vs intermediate or high), and adulthood socioeconomic status (low vs intermediate or high; not available in HeSSup). Additional stratifications were based on lifestyle and clinical factors: smoking (current vs former or never), physical activity (low vs moderate or recommended), and alcohol consumption (heavy vs none or moderate drinking). Health-related subgroups included stratification by use of glucocorticoids (yes vs no), hypertension (yes vs no), metabolic syndrome (yes vs no), depression (yes vs no), and major chronic diseases: cardiometabolic disease (type 1 or 2 diabetes, coronary heart disease or stroke vs none), diabetes (yes vs no), respiratory disease (asthma or chronic obstructive pulmonary disease (COPD) vs none), cancer (yes vs no), and any baseline chronic disease (yes vs no). Subgroup differences were tested using heterogeneity test. The proportion of participants with missing data for baseline covariates was relatively small, ranging between 0% and 8.0% (**table 1**). Therefore, participants with missing data were excluded from the corresponding subgroup analyses.

Due to the smaller number of cases in analyses of specific infection types, we used fixed effects meta-analysis to calculate pooled effect estimates across the Finnish cohorts and UK Biobank.

Analysis of GBD data. To estimate the infection burden attributable to obesity, assuming that the observed associations were causal, we computed population attributable fraction (PAF) for obesity using the formula:

$$[(p * (RR - 1)) / ((p * (RR - 1)) + 1)] * 100\%$$

where p is the proportion of individuals with obesity in the population and RR the relative risk of infectious death for individuals with obesity compared with those without obesity. For these calculations, we used the weighted average HR for infection-related deaths (1.56, 95% CI 1.48–

1.63) across the primary and replication analyses, given the homogeneity of HRs across infection types and data sources. For analyses of 2021 and 2023 data, we used separate HRs for COVID-19 infectious deaths (2.19, 95% CI 1.98–2.43).

To estimate the infection burden attributable to obesity more widely, we used obesity prevalence estimates and infectious disease deaths from 2023 (https://ghdx.healthdata.org/gbd-2023), the latest year with Global Burden of Disease (GBD) data available across all regions and countries through the Data Sources Tool (https://vizhub.healthdata.org/gbd-results/). A Monte Carlo simulation with 1,000 iterations was conducted to estimate 95% confidence intervals for PAFs. In each iteration, values were randomly sampled from the distributions of obesity prevalence, infectious disease deaths (number or rate), and the hazard ratio (HR) for infectious disease mortality associated with obesity, based on their point estimates, and standard errors and entered in the PAF formula. The median and the 2.5^{th} and 97.5^{th} percentiles of the distribution of infectious disease deaths attributable to obesity obtained from 1000 simulations were used as the point estimate and the bounds of the 95% confidence interval.

Statistical software: Analyses were conducted using SAS 9·4, RStudio 2025.09.1 Build 401, and Stata 19·5 statistical software.

2 Supplementary Results

Baseline characteristics of the participants and the number of incident severe infection cases in the Finnish dataset and UK Biobanks are provided in the paper (**table 1**). Mean age at the assessment of BMI and at incident infection is shown in **table S2**.

Of the 67 766 participants in the Finnish dataset, 1 493 had diabetes at baseline (prevalence 2·2%). In UK Biobank (N=479 498), 24 373 participants (5·1%) had diabetes. Individuals with diabetes were overrepresented among those who died from infectious causes. In the Finnish dataset, 287 participants died from an infectious disease, of whom 29 (10·1%) had diabetes at baseline. In UK Biobank, there were 7 479 infectious deaths, including 1 119 (15·0%) among participants with diabetes.

Table S2. Mean age at the assessment of BMI and at incident infection

	**Mean (SD) age**	
	**Finnish dataset**	**UK Biobank**
Mean age of BMI measurement	42·1 (10·8)	57·0 (8·1)
Mean age at hospitalisation or death due to infection:		
Any infection	50.7 (12.7)	67.0 (8.6)
By type		
Bacterial infection	50.9 (12.7)	67.1 (8.6)
Viral infection	49.1 (13.5)	68.9 (8.6)
Parasitic infection	53.3 (11.9)	67.0 (8.5)
Fungal infection	56.6 (11.4)	69.1 (7.8)
By chronicity		
Acute infection	50.9 (12.7)	67.3 (8.6)
Chronic infection	49.8 (13.0)	64.0 (9.1)
Common infections		
Acute pharyngitis or acute tonsillitis	38.7 (12.3)	63.6 (9.1)
Influenza	54.5 (13.3)	68.5 (8.3)
Pneumonia	54.8 (11.6)	69.6 (7.7)
Lower respiratory tract infection	54.8 (11.6)	69.0 (7.9)
Urinary tract infection	55.8 (11.9)	69.2 (8.1)
Gastrointestinal infection	50.6 (12.5)	68.0 (8.4)
Skin or soft tissue infection	53.3 (11.5)	66.6 (8.9)
HIV infection	47.1 (12.5)	62.5 (10.7)
Tuberculosis	56.5 (10.4)	64.8 (8.6)
COVID-19	–	72.4 (7.7)

Obesity-severe infection association: There was no evidence of heterogeneity in the cohort-specific age- and sex-adjusted hazard ratios for obesity versus healthy weight and risk of severe infection between the two Finnish cohorts (HR 1·56, 95% CI 1.44–1.68 in FPS; HR 1.69, 95% CI 1.51–1.89 in HeSSup; I^2=28.3 %; p=0·237), supporting the pooling of these datasets (HR 1.59, 95% CI 1.50–1. 70 based on pooled data, HR 1.60, 95% CI 1.50–1. 70 in fixed-effect meta-analysis). The corresponding HR was 1.74, 95% CI 1.71–1.77 in UK Biobank and 1.73, 95% CI 1.70–1.76 across all cohorts.

Similarly, we observed little heterogeneity by ethnic background, the hazard ratio being HR 1.73, 95% CI 1.70—1.77 in White, HR 1.91, 95% CI 1.68—2.18 in Asian, HR 1.40, 95% CI 1.20—1.64 in Black, and HR 1.87, 95% CI 1.62—2.15 in all other ethnicities.

The proportion of missing data for each covariate is shown in **table 1**. **Figure S2** presents hazard ratios for BMI categories in the basic model and after adjustment for individual covariates and for all covariates combined, separately for the Finnish dataset and UK Biobank. The graded association between higher BMI category and higher risk of severe infection persisted across all adjustment models. The age- and sex-adjusted hazard ratio for class III obesity versus healthy weight was 2.7 (2.2–3.3) in the Finnish dataset and 3.1 (3.0–3.2) in UK Biobank. These associations remained after multivariable adjustments (2.2, 1.8–2.7; and 2.0, 1.9–2.1, respectively), and remained similar when analyses were repeated following multiple imputation for missing covariates (2.2, 1.7–2.6; and 2.0, 1.9–2.0).

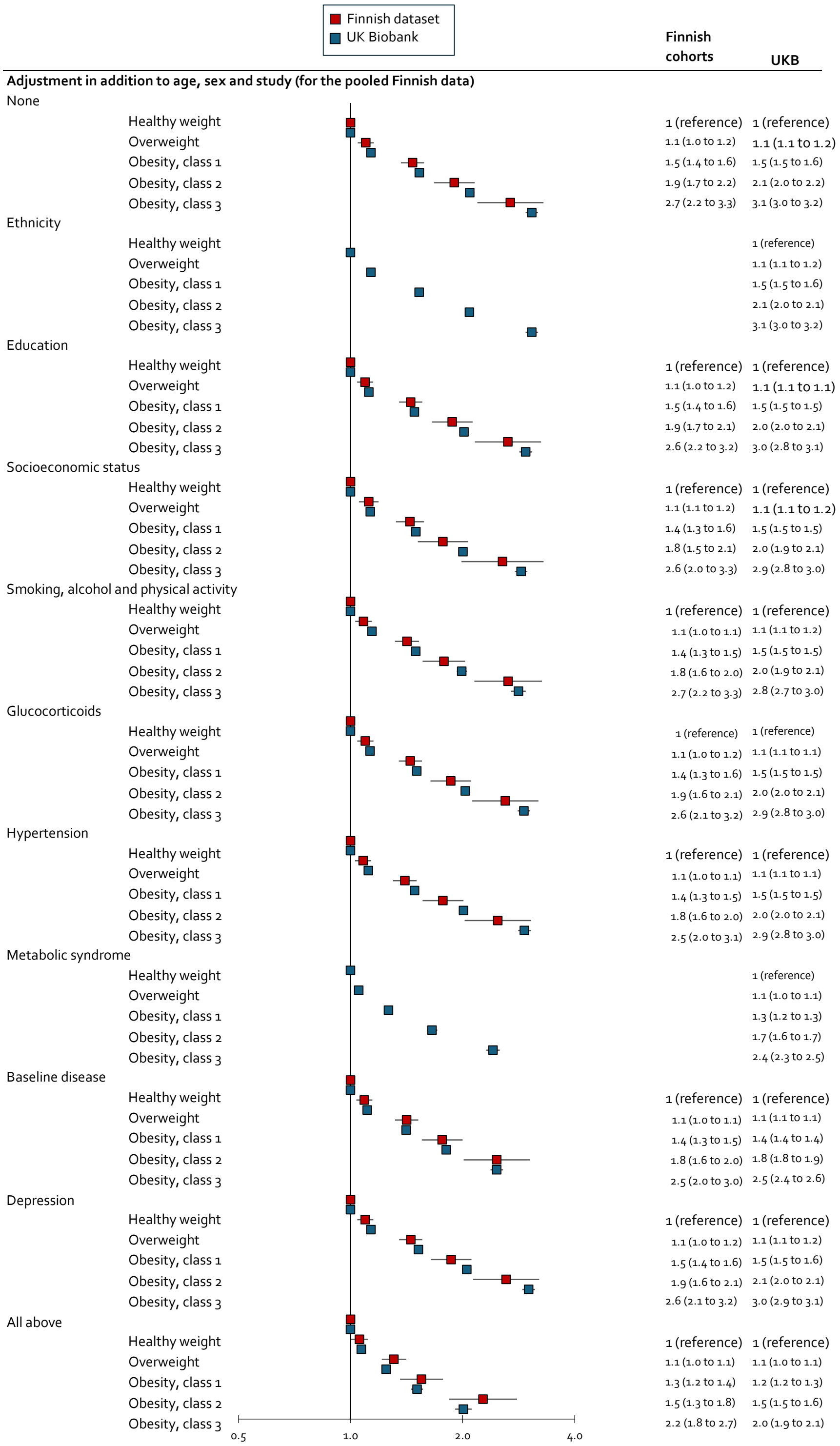

Figure S2. Association between BMI category and risk of severe infectious disease with different adjustments in the Finnish cohorts and UK Biobank

We found no evidence of bias due to competing risk by mortality. The age- and sex-adjusted hazard ratio for obesity versus healthy weight in relation to incident infection risk was 1.59 (95% CI 1.50—1.70) in the Finnish dataset and 1.74 (95% CI 1.71— 1.77) in UK Biobank. Corresponding estimates from Fine and Gray competing risk models were 1.58 (95% CI 1.48—1.68) and 1.73 (95% CI 1.69—1.76), respectively (**table S3**).

Table S3. Association between obesity and risk of severe infection based on Fine and Gray regression in Finnish cohorts and UK Biobank

BMI category	N(total)	N(event)	HR	95% confidence interval	
**Finnish dataset**					
Healthy weight	39156	4264	1.00		
Overweight	21216	2667	1.10	1.05	1.16
Obesity	7394	1299	1.58	1.48	1.68
N total	67766	8230			
**UK Biobank**					
Healthy weight	157 917	20 976	1.00		
Overweight	205 319	33 881	1.14	1.12	1.16
Obesity	83 797	17 892	1.73	1.69	1.76
N total	23 567	6220			

To assess whether changes in BMI after baseline influenced the effect estimates, we incorporated repeated BMI measurements during follow-up into age- and sex-adjusted Cox models as time-dependent covariates. In the Finnish dataset, where repeated measurements were available, hazard ratios for obesity were similar in models using both two and four BMI assessments (**table S4**).

Table S4. Association between obesity and risk of severe infection in Finnish cohorts using repeat BMI assessments

	HR	95% CI	
**BMI assessment at baseline and once during follow-up**			
Healthy weight	1.00		
Overweight	1.09	1.03	1.14
Obesity	1.56	1.47	1.66
**BMI assessment at baseline and 3 times during follow-up**			
Healthy weight	1.00		
Overweight	1.05	1.00	1.10
Obesity	1.54	1.46	1.64

Furthermore, adult obesity was associated with risk of recurrent severe infections as indicated by Cox proportional hazards regression models with the second severe infection as the outcome (**table S5**).

Table S5. Association between obesity and risk of recurrent severe infections

BMI category	N(total)	N(event)	HR	95% CI	
**Finnish dataset**					
Healthy weight	39156	1103	1.00		
Overweight	21216	772	1.09	1.00	1.20
Obesity	7394	458	1.91	1.71	2.14
Total number	67766	2333			
**UK Biobank**					
Healthy weight	157 917	6400	1.00		
Overweight	205 319	10 951	1.14	1.10	1.17
Obesity	116 262	10 062	1.99	1.93	2.05
Total number	479 498	27 413			

Change in BMI: In the Finnish data, changes in BMI category were associated with the risk of severe infections in both absolute and relative terms. The lowest incidence was observed among those with persistent healthy weight (73.3 per 10 000 person-years) and the highest among those with persistent obesity (141·7 per 10 000 person-years) (**figure S3**). Compared with persistent obesity, weight loss from obesity to overweight or healthy weight reduced risk (HR 0·8, 95% CI: 0·6 to 1·0, incidence 111.1 per 10 000 person-years), although not to the level of the persistent healthy weight group. Weight gain from overweight to obesity increased risk 1.3 times (1.1 to 1.5, incidence 109.4 per 10 000 person-years), but not to the level observed in persistent obesity. When compared with persistent healthy weight, HR was 1·1 (1·0 to 1·2) for weight gain to overweight or obesity.

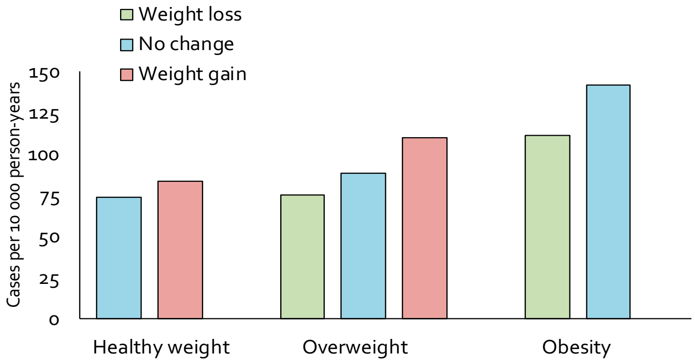

Change in BMI category		Hazard ratios (95% CI) for incident infection after Wave 2
Wave 1	Wave 2	
Healthy weight	Healthy weight	1·0 (reference)
	Overweight or obesity	1·1 (1·0 to 1·2) – weight gain
Overweight	Overweight	1·0 (reference)
	Healthy weight	0·9 (0·7 to 1·1) – weight loss
	Obesity	1·3 (1·1 to 1·5) – weight gain
Obesity	Obesity	1·0 (reference)
	Overweight or healthy weight	0·8 (0·6 to 1·0) – weight loss

Figure S3. Association of change in BMI category with risk of incident severe infection in the Finnish cohorts

Subgroup analysis. As shown in **figure 3**, **table S6**, and **figure S4**, subgroup differences in the obesity–infection association (p<0·0001 and HR difference >0·3) were observed for five factors, with lower hazard ratios among smokers than non-smokers (1·3 vs 1·9), those with low versus high physical activity (1·5 vs 1·8), participants using glucocorticoid medication (1·4 vs 1·7), individuals with chronic disease (1·3 vs 1·6), and those with respiratory disease (1·4 vs 1·7). Subgroup differences for overweight were smaller (**table S7**).

Table S6. Subgroup differences in the association between obesity vs healthy weight and risk of severe infections by baseline covariates

Subgroup	Q stat	P-value	I^2
Sex (men vs women)	21.92	<0.0001	95.40 %
Age <40 vs 40+	0.25	0.62	0.00 %
Age <50 vs 50+	30.03	<0.0001	96.70 %
Low vs high education	73.10	<0.0001	98.60 %
Low vs high SES	4.00	0.045	75.00 %
White vs non-White ethnicity	0.17	0.68	0.00 %
Smoking (yes vs no)	247.38	<0.0001	99.60 %
Physical inactivity (yes vs no)	66.38	<0.0001	98.50 %
Heavy drinking (yes vs no)	48.72	<0.0001	97.90 %
Use of glucocorticoids (yes vs no)	42.19	<0.0001	97.60 %
Hypertension (yes vs no)	2.98	0.084	66.40 %
Metabolic syndrome (yes vs no)	23.82	<0.0001	95.80 %
Depression (yes vs no)	1.89	0.17	47.00 %
Any chronic disease (yes vs no)	108.49	<0.0001	99.10 %
Diabetes (yes vs no)	4.80	0.028	79.20 %
Cardiometabolic disease (yes vs no)	13.33	<0.0001	92.50 %
Respiratory disease (yes vs no)	34.17	<0.0001	97.10 %
Cancer (yes vs no)	31.97	<0.0001	96.90 %

Table S7. Subgroup differences in the association between overweight vs healthy weight and risk of severe infections by baseline covariates

Subgroup	Q stat	P-value	I^2
Sex (men vs women)	33.61	<0.0001	97.00 %
Age <40 vs 40+	1.36	0.24	26.70 %
Age <50 vs 50+	6.64	0.01	84.90 %
Low vs high education	43.02	<0.0001	97.70 %
Low vs high SES	7.54	0.006	86.70 %
White vs non-White ethnicity	2.63	0.105	62.00 %
Smoking (yes vs no)	99.4	<0.0001	99.00 %
Physical inactivity (yes vs no)	42.41	<0.0001	97.60 %
Heavy drinking (yes vs no)	29.35	<0.0001	96.60 %
Use of glucocorticoids (yes vs no)	15.36	<0.0001	93.50 %
Hypertension (yes vs no)	4.6	0.032	78.30 %
Metabolic syndrome (yes vs no)	12.52	<0.0001	92.00 %
Depression (yes vs no)	0.05	0.82	0.00 %
Any chronic disease (yes vs no)	27.66	<0.0001	96.40 %
Diabetes (yes vs no)	0.02	0.88	0.00 %
Cardiometabolic disease (yes vs no)	1.53	0.22	34.70 %
Respiratory disease (yes vs no)	15.75	<0.0001	93.70 %
Cancer (yes vs no)	5.65	0.017	82.30 %

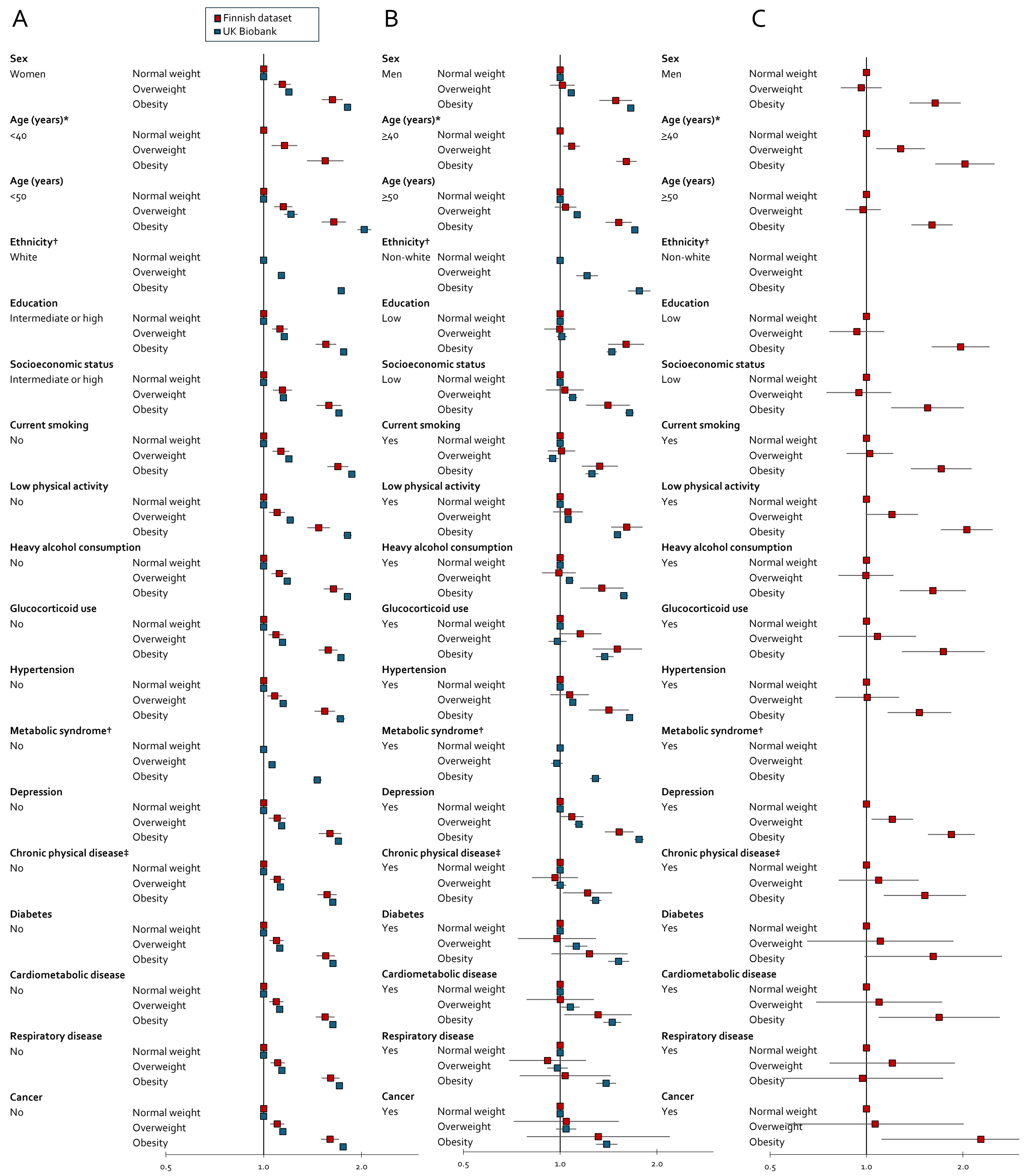

Figure S4. Association between BMI category and risk of severe infections in subgroups

A) Individuals without a baseline risk factor, B) with a risk factors at baseline, or C) additionally with the same BMI category at both baseline and after 4-5 years.

* Only Finnish cohorts

† Only UK Biobank

‡ Diabetes, coronary heart disease, stroke, asthma, chronic obstructive pulmonary disease, or cancer.

The role of diabetes and use of glucocorticoids. The age- and sex-adjusted hazard ratio for the association between obesity and risk of severe infections in individuals with comorbid diabetes was 1.55 (95% CI 1.45—1.66) in the Finnish dataset and 1.52 (95% CI 1.41—1.64) in UK Biobank, values that were close to the corresponding hazard ratios observed for all participants 1.59 (95% CI 1.50—1.70) and 1.74 (95% CI 1.71— 1.77) , respectively). Similarly, the association was also observed among participants treated with glucocorticoids, a medication that affects immune function (**table S8**). Among the 4867 glucocorticoid users in the Finnish dataset, the incidence of severe infections was 18·2% (886 cases), compared with 11·7% among non-users (N=62 899; 7344 cases). In UK Biobank, 18 832 participants used glucocorticoids, among whom 6480 severe infections occurred (34·4%), compared with 16·4% (75 465 of 460 666) among non-users.

Table S8. Association between BMI category and risk of severe infectious disease by use of glucocorticoids medication

	No glucocorticoid use					Glucocorticoid use				
**BMI**	**N(total)**	**N(event)**	**HR**	**95% CI**		**N(total)**	**N(event)**	**HR**	**95% CI**	
**Finnish dataset**										
Healthy weight	36 592	3856	1.00			2564	408	1.00		
Overweight	19 661	2371	1.09	1.04	1.15	1555	296	1.16	0.99	1.34
Obesity	6646	1117	1.58	1.48	1.69	748	182	1.51	1.26	1.80
Total number	62 899	7344				4867	886			
**UK Biobank**										
Healthy weight	153 025	19478	1.00			4892	1498	1.00		
Overweight	197 777	31467	1.14	1.12	1.16	7542	2414	0.98	0.92	1.05
Obesity	109 864	24520	1.73	1.70	1.76	6398	2568	1.38	1.29	1.47
Total number	460 666	75465				18832	6480			

Alternative adiposity indicators. Comparison of alternative measures of obesity showed that the association with severe infections was robust to the choice of definition (**table S9**). HRs for obesity versus healthy weight were consistent across BMI (1.7, 95% CI 1.7 – 1.8), waist circumference (1.7, 1.7 – 1.8), waist-to-height ratio (2.1 ,2.0 – 2.1), and the combined obesity measure (2.1 ,2.1–2.2). The associations with bacterial, viral, parasitic and fungal infections were also consistent.

Table S9. Association between adiposity category and risk of severe infectious disease stratified by method of assessment in UK Biobank

	Hazard ratio (95% CI) for obesity vs healthy weight by assessment method*			
**Different infections**	**BMI**	**Waist**	**Waist-to-height ratio**	**Combined measure†**
Any	1.7 (1.7 to 1.8)	1.7 (1.7 to 1.8)	2.1 (2.0 to 2.1)	2.1 (2.1 to 2.2)
Bacterial infections	1.7 (1.7 to 1.8)	1.7 (1.7 to 1.8)	2.1 (2.0 to 2.1)	2.1 (2.1 to 2.2)
Viral infections	2.1 (2.0 to 2.3)	2.1 (1.9 to 2.2)	2.7 (2.5 to 2.9)	2.8 (2.6 to 3.0)
Parasitic infections	1.6 (1.2 to 2.2)	1.6 (1.2 to 2.2)	1.9 (1.3 to 2.7)	2.1 (1.4 to 3.1)
Fungal infections (mycoses)	1.6 (1.5 to 1.7)	1.7 (1.5 to 1.8)	2.1 (1.9 to 2.3)	2.0 (1.8 to 2.3)

Spline models of adiposity indicators demonstrated relatively consistent associations between continuous measures of BMI, waist circumference, and waist-to-height ratio and the risk of severe infections (**figure S5**).

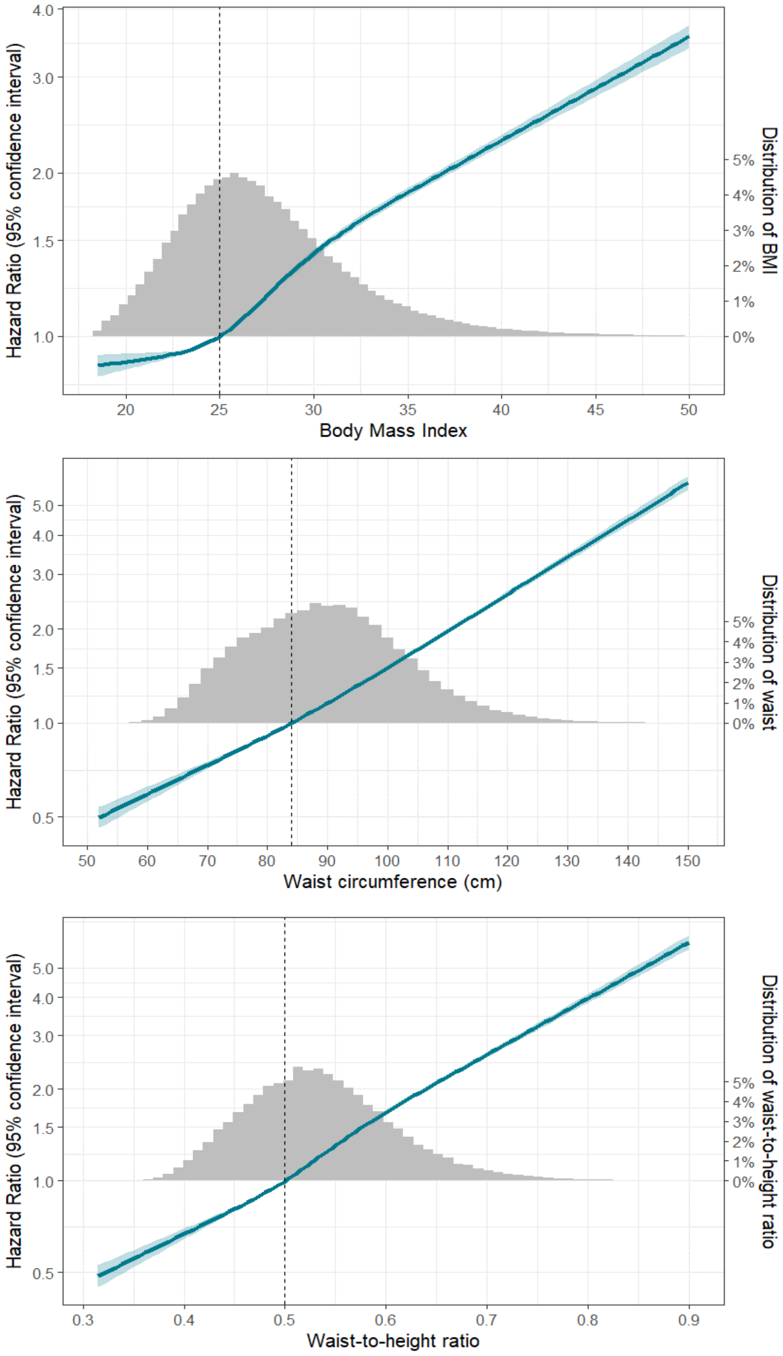

Figure S5. Associations between continuous adiposity indicators and risk of severe infectious disease

Vertical line indicates the reference value: the upper threshold for healthy weight according to BMI (25 kg/m²), and the equivalent 33% percentile in the other adiposity indicators. Participants with BMI <18·5 kg/m² were excluded from the study population.

Associations by infection type and chronicity, and for specific infections. **Figure S6** shows the association between obesity and risk of severe infections by type, chronicity, and cohort, and **figures S7 and S8** present corresponding associations for subcategories of bacterial and viral infections and for specific infectious diseases.

Infection category	Total	Events	Finnish data	UKB
Any infection	67766	8230	1.6 (1.5 to 1.7)	1.7 (1.7 to 1.8)
	479498	81945		
**By type**				
Bacterial infections	67766	7598	1.6 (1.5 to 1.7)	1.7 (1.7 to 1.8)
	479498	78412		
Viral infections	67766	732	1.4 (1.1 to 1.7)	2.1 (2.0 to 2.3)
	479498	7357		
Parasitic infections	67766	28	1.6 (0.6 to 4.5)	1.6 (1.2 to 2.2)
	479498	280		
Fungal infections (mycoses)	67766	56	2.4 (1.2 to 4.8)	1.6 (1.5 to 1.7)
	479498	3573		
**By chronicity**				
Acute	67766	7869	1.6 (1.5 to 1.7)	1.8 (1.7 to 1.8)
	479498	77815		
Chronic	67766	600	1.5 (1.2 to 1.9)	1.6 (1.5 to 1.7)
	479498	6910		

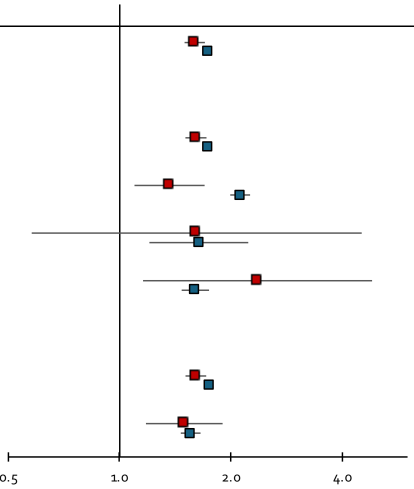

Figure S6. Association between obesity and risk of severe infections by type, chronicity and cohort

Bacterial infections	Total	Events	Finnish data	UKB
Mycoplasma infections	67766	13	3.0 (0.8 to 10.6)	1.8 (0.9 to 4.0)
	479498	51		
Gram-positive bacterial infections	67766	413	2.2 (1.7 to 2.9)	2.0 (1.9 to 2.2)
	479498	4905		
Bacterial infections with sepsis	67766	563	1.9 (1.6 to 2.4)	2.0 (1.8 to 2.1)
	479498	5918		
Localised bacterial infections	67766	2236	1.9 (1.7 to 2.2)	1.9 (1.8 to 1.9)
	479498	54217		
Extracellular bacterial infections	67766	498	1.9 (1.5 to 2.4)	1.9 (1.8 to 2.0)
	479498	10882		
Chronic bacterial infections	67766	359	1.7 (1.2 to 2.2)	1.7 (1.5 to 1.8)
	479498	5621		
Bacterial infections without sepsis	67766	7403	1.6 (1.5 to 1.7)	1.7 (1.7 to 1.8)
	479498	77552		
Acute bacterial infections	67766	7375	1.6 (1.5 to 1.7)	1.7 (1.7 to 1.8)
	479498	74998		
Invasive bacterial infections	67766	5980	1.6 (1.5 to 1.7)	1.7 (1.6 to 1.7)
	479498	43036		
Gram-negative bacterial infections	67766	392	1.2 (0.9 to 1.7)	2.0 (1.9 to 2.1)
	479498	8030		
Intracellular bacterial infections	67766	158	1.1 (0.7 to 1.9)	1.1 (0.8 to 1.5)
	479498	240		
Mycobacterial infections	67766	36	0.8 (0.3 to 2.2)	0.7 (0.4 to 1.1)
	479498	122		
**Viral infections**				
Acute viral infections	67766	514	1.4 (1.1 to 1.8)	2.3 (2.2 to 2.5)
	479498	6412		
Herpesvirus (persistent infections)	67766	138	1.6 (1.0 to 2.5)	1.2 (1.0 to 1.5)
	479498	868		
Persistent infection (other that herpesvirus)	67766	100	0.8 (0.4 to 1.7)	0.9 (0.6 to 1.2)
	479498	296		

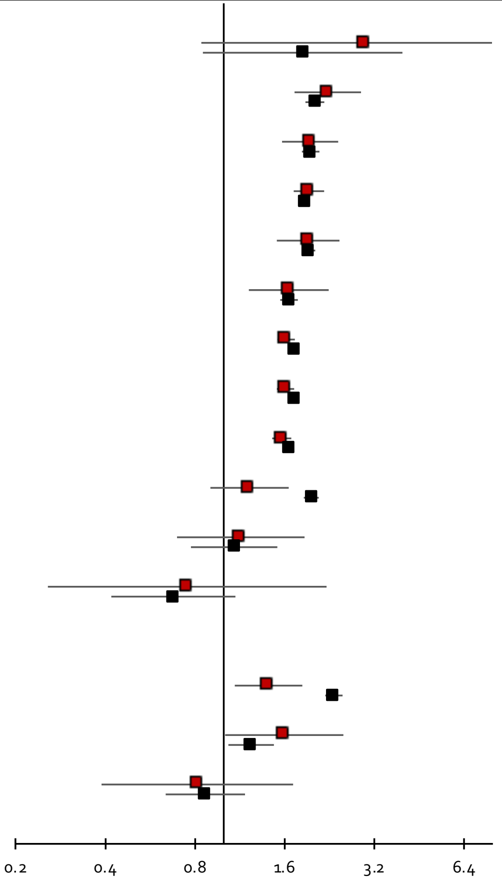

Figure S7. Association between obesity and risk of severe bacterial and viral infections by subtype and cohort

Infection category	Total	Events	Finnish data	UKB
Skin and soft tissue infection	67766	1197	4.0 (3.5 to 4.7)	2.7 (2.5 to 2.8)
	479498	14972		
Covid-19 (UKB only)	-	-	-	2.3 (2.1 to 2.4)
	479498	7985		
Gastrointestinal infection	67766	781	1.7 (1.4 to 2.1)	2.1 (1.8 to 2.4)
	479498	1682		
Urinary tract infection	67766	930	1.7 (1.5 to 2.1)	1.8 (1.7 to 1.8)
	479498	23596		
Lower respiratory tract infection	67766	2240	1.5 (1.3 to 1.6)	1.8 (1.7 to 1.8)
	479498	38620		
Influenza	67766	102	1.4 (0.8 to 2.5)	1.7 (1.5 to 1.9)
	479498	1566		
Pneumonia	67766	2038	1.4 (1.2 to 1.6)	1.7 (1.6 to 1.7)
	479498	28647		
Acute pharyngitis or tonsillitis	67766	159	2.1 (1.3 to 3.3)	1.4 (1.3 to 1.6)
	479498	1580		
HIV	67766	<10	3.6 (0.6 to 22.5)	0.6 (0.3 to 1.5)
	479498	<30		
Tuberculosis	67766	28	0.7 (0.2 to 2.4)	0.7 (0.4 to 1.2)
	479498	85		

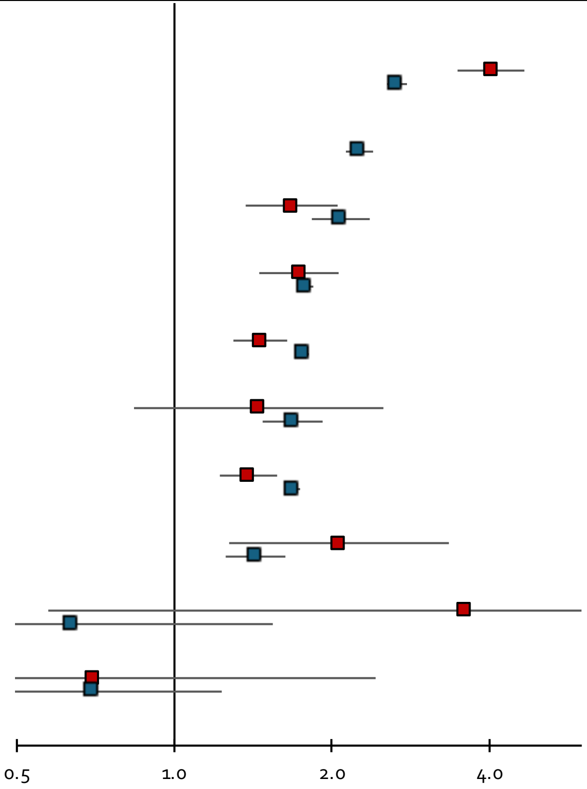

Figure S8. Association between obesity and risk of selected severe infections by cohort

Table S9 compares results between the two datasets for the association between obesity and risk of severe infection by type and for specific infections. With the exception of HIV, tuberculosis and parasitic infections, all associations were statistically significant in both the Finnish cohorts and the UK Biobank. The associations with HIV and tuberculosis were not significant in either dataset. The association with parasitic infections was statistically significant in the UK Biobank only, but the HR was 1.6 in both datasets. These findings support the consistency of results across cohorts. The lack of association for HIV and tuberculosis might be partly explained by reverse causation bias, given the wasting effects characteristic of these infections and the prominent role of weight loss in disease progression.

The strongest associations were observed for skin and soft tissue infections, with obesity associated with more than a twofold increase in risk in both datasets and in both minimally adjusted and fully adjusted models (**tables S9 and S10**). The relatively small attenuation after multivariable adjustment further supports the robustness of these findings and suggests a low risk of confounding.

Data on COVID-19 were available only from the UK Biobank. Obesity doubled the relative risk of COVID-19, with adjustment for baseline covariates—including both confounders and potential mediators—attenuating the estimate to 1·5 (**table S10**). The corresponding relative risks were lower for gastrointestinal infections, urinary tract infections, lower respiratory tract infections, influenza, pneumonia, and acute pharyngitis or tonsillitis, but remained statistically significant.

Table S9. Association between obesity and risk of severe infectious disease by type and for selected infections in the Finnish cohorts and UK Biobank

Outcome	Finnish data	UK Biobank
**Any infection**	1.6 (1.5 to 1.7)	1.7 (1.7 to 1.8)
**By type**		
Bacterial infections	1.6 (1.5 to 1.7)	1.7 (1.7 to 1.8)
Viral infections	1.4 (1.1 to 1.7)	2.1 (2.0 to 2.3)
Parasitic infections	1.6 (0.6 to 4.5)	1.6 (1.2 to 2.2)
Fungal infections (mycoses)	2.4 (1.2 to 4.8)	1.6 (1.5 to 1.7)
**Selected infections**		
Skin and soft tissue infection	4.0 (3.5 to 4.7)	2.7 (2.5 to 2.8)
COVID-19	N/A	2.3 (2.1 to 2.4)
Gastrointestinal infection	1.7 (1.4 to 2.1)	2.1 (1.8 to 2.4)
Urinary tract infection	1.7 (1.5 to 2.1)	1.8 (1.7 to 1.8)
Lower respiratory tract infection	1.5 (1.3 to 1.6)	1.8 (1.7 to 1.8)
Influenza	1.4 (0.8 to 2.5)	1.7 (1.5 to 1.9)
Pneumonia	1.4 (1.2 to 1.6)	1.7 (1.6 to 1.7)
Acute pharyngitis or tonsillitis	2.1 (1.3 to 3.3)	1.4 (1.3 to 1.6)
HIV	3.6 (0.6 to 22.5)	0.6 (0.3 to 1.5)
Tuberculosis	0.7 (0.2 to 2.4)	0.7 (0.4 to 1.2)

Hazard ratios for obesity versus healthy weight are adjusted for age and sex.

Table S10. Comparison of the basic model and multivariable-adjusted model of obesity and risk of severe infectious disease

Outcome	Age and sex adjusted	Multivariable adjusted
**Any infection**	1.7 (1.7 to 1.8)	1.3 (1.3 to 1.4)
**By type**		
Bacterial infections	1.7 (1.7 to 1.8)	1.3 (1.3 to 1.4)
Viral infections	2.0 (1.9 to 2.2)	1.5 (1.3 to 1.6)
Parasitic infections	1.6 (1.2 to 2.2)	1.1 (0.8 to 1.7)
Fungal infections (mycoses)	1.6 (1.5 to 1.8)	1.1 (1.0 to 1.2)
**Selected infections**		
Skin and soft tissue infection	2.8 (2.6 to 2.9)	2.1 (2.0 to 2.2)
Covid-19 (UKB only)	2.3 (2.1 to 2.4)	1.5 (1.4 to 1.7)
Gastrointestinal infection	2.0 (1.8 to 2.2)	1.3 (1.1 to 1.5)
Urinary tract infection	1.8 (1.7 to 1.8)	1.3 (1.2 to 1.3)
Lower respiratory tract infection	1.7 (1.7 to 1.8)	1.3 (1.2 to 1.3)
Influenza	1.7 (1.5 to 1.9)	1.2 (1.0 to 1.4)
Pneumonia	1.7 (1.6 to 1.7)	1.2 (1.2 to 1.3)
Acute pharyngitis or tonsillitis	1.5 (1.3 to 1.7)	1.3 (1.1 to 1.5)
HIV	0.9 (0.4 to 2.0)	0.9 (0.3 to 2.5)
Tuberculosis	0.7 (0.4 to 1.2)	0.7 (0.3 to 1.3)

*Multivariable model is adjusted for age, sex, ethnicity, education, socioeconomic status, smoking, alcohol consumption, physical activity, glucocorticoid medication, hypertension, metabolic syndrome, chronic diseases (diabetes, coronary heart disease, stroke, asthma, COPD, cancer) and depression

3 Global and Regional Impact of Obesity on Infectious Deaths

We provide worldwide estimates of infectious deaths attributable to adult obesity, as well as estimates by super-regions, regions, and countries, for three time points—2018 (pre-pandemic), 2021 (during the pandemic), and 2023 (post-pandemic/post emergency period, the most recent data available)—in **appendix 3**.

4 Statistical Syntax

```
*Basic and adjusted models, covariates added as appropriate;
proc phreg data=fin_inf; class sex wgc(ref="0") study;
model fup_i999*status_i999(0)= age sex wgc study /rl;
ods output CensoredSummary=m1n ParameterEstimates=m1e;
run;

#R-version
library(survival)[40]
  model1 = Surv(futime_dg999, status_dg999) ~ age + as.factor(sex) + wgcl2


*Subgroup models;
proc sort data=fin_inf; by sex; run;
proc phreg data=fin_inf; by sex; class study obe(ref="0");
model fup_i999*status_i999(0)= age obe study/rl;
ods output CensoredSummary=sex_999_n ParameterEstimates=sex_999_est; run;

# R-version
    cox_model <- coxph(Surv(futime_dg999, status_dg999) ~ age + as.factor(sex) +
as.factor(obe), data = condition)

*Time-dependent obesity*;
proc phreg data=inf3;
class sex study;
model fupy_i999*status_i999(0)= age sex study obetime1 obetime2 /rl;
obetime1=obe1; obetime2=obe2;
if obe04>. and fupy_i999>=fubmi04 then do; obetime1=obe1_04; obetime2=obe2_04;
end;
if obe08>. and fupy_i999>=fubmi08 then do; obetime1=obe1_08; obetime2=obe2_08;
end;
if obe12>. and fupy_i999>=fubmi12 then do; obetime1=obe1_12; obetime2=obe2_12;
end;
run;

*competing risk by mortality*;
proc phreg data=inf1;
class sex study obe(ref='0');
model fup_i999*status3_i999(0)= age sex study obe / eventcode=2 rl;
run;
```

*Example code for meta-analysis (Stata)
metan esti sele if (type=="Any"), fixed label(namevar=Inftype) lcols("Inftype") t1title ("Any infection") eform

Population attributable death rates for obesity
Author: Sara Ahmadi-Abhari

```
clear
set more off
```

```
local dataset "data containing prevalence and death rates by location"

use "`dataset'", clear

********** Create variables for Population attributable rates (/number) and
fractions.

gen par_total=.
gen lower95_total=.
gen upper95_total=.

gen par_covid=.
gen lower95_covid=.
gen upper95_covid=.

gen par_nonCovid=.
gen lower95_nonCovid=.
gen upper95_nonCovid=.

gen paf_total=.
gen paf_lower95_total=.
gen paf_upper95_total=.

gen paf_covid=.
gen paf_lower95_covid=.
gen paf_upper95_covid=.

gen paf_nonCovid=.
gen paf_lower95_nonCovid=.
gen paf_upper95_nonCovid=.

save "`dataset'", replace

local n_regions = _N


*------------------------------
* Monte Carlo settings
*------------------------------

local n_iter = 1000

*------------------------------
* Prepare temporary iterations storage dataset
*------------------------------

tempfile iterations
save `iterations', replace

*------------------------------
* Loop over each region
*------------------------------
use `iterations', clear

forvalues i = 1/`n_regions' {

    preserve
    * Load parameters for this region
      use "`dataset'", clear
    keep in `i'
    local prev = obesity_prev[1]
```

```
    local se_prev = obesity_prev_se[1]

      local death_ln_inf=rate_inf_deaths_ln[1] /// *** logarithm of death rates
from all infectious diseases
      local se_death_ln_inf=rate_inf_deaths_ln_se[1] /// *** standard error of
logarithm of death rates from all infectious diseases

      local death_ln_covid=rate_covid_ln[1] /// *** logarithm of COVID death
rates
    local se_death_ln_covid =rate_covid_ln_se[1] /// *** standard error of
logarithm of COVID death rates

      local death_ln_nonCovid=rate_nonCovid_ln[1] /// *** logarithm of non-COVID
death rates
    local se_death_ln_nonCovid =rate_nonCovid_ln_se[1] /// *** standard error of
logarithm of non-COVID death rates


      *RR of non-COVID deaths for obesity
      local rr_nonCovid= *** input log(RR) from meta-analysis
      local se_rr_nonCovid= *** input standard error of log(RR) from meta-
analysis
      *RR of COVID deaths for obesity
      local rr_covid= *** input log(RR)
      local se_rr_covid= *** input standard error of log(RR)



    *------------------------------
    * Create Monte Carlo dataset for this region
    *------------------------------
    clear
    set obs `n_iter'

    * 1. Simulate prevalence (resample if out of bounds)
    gen prev_sim = rnormal(`prev', `se_prev')
    local iteration = 0
    while (1) {
        local iteration = `iteration' + 1
        quietly count if prev_sim < 0 | prev_sim > 1
        local n_bad = r(N)
        if (`n_bad' == 0) continue, break
        replace prev_sim = rnormal(`prev', `se_prev') if prev_sim < 0 | prev_sim
> 1
        if (`iteration' > 100) continue, break
    }

          * 2. Simulate deaths

      gen death_sim_ln_inf = rnormal(`death_ln_inf', `se_death_ln_inf')
    gen death_sim_inf=exp(death_sim_ln_inf)

      gen death_sim_ln_covid = rnormal(`death_ln_covid', `se_death_ln_covid')
    gen death_sim_covid=exp(death_sim_ln_covid)

      gen death_sim_ln_nonCovid = rnormal(`death_ln_nonCovid',
`se_death_ln_nonCovid')
    gen death_sim_nonCovid=exp(death_sim_ln_nonCovid)


    * 3. Simulate RR (log-normal)
```

```
    gen logrr_sim_covid = rnormal(`rr_covid', `se_rr_covid')
    gen rr_sim_covid = exp(logrr_sim_covid)

      gen logrr_sim_nonCovid = rnormal(`rr_nonCovid', `se_rr_nonCovid')
    gen rr_sim_nonCovid = exp(logrr_sim_nonCovid)


    * 4. Compute population attributable death rates for obesity

      gen par_sim_covid=death_sim_covid*((prev_sim)*(rr_sim_covid-
1))/(1+((prev_sim)*(rr_sim_covid-1)))

      gen par_sim_nonCovid=death_sim_nonCovid*((prev_sim)*(rr_sim_nonCovid-
1))/(1+((prev_sim)*(rr_sim_nonCovid-1)))

      egen par_sim_total= rowtotal(par_sim_covid par_sim_nonCovid)

      gen paf_total=100*par_sim_total/death_sim_inf
      gen paf_covid=100*par_sim_covid/death_sim_covid
      gen paf_nonCovid=100*par_sim_nonCovid/death_sim_nonCovid


    * 5. Summarize Monte Carlo results

      centile par_sim_total, centile(2.5 50 97.5)
    scalar par_l_total = r(c_1)
      scalar par_m_total = r(c_2)
    scalar par_u_total = r(c_3)

    centile par_sim_covid, centile(2.5 50 97.5)
    scalar par_l_covid = r(c_1)
      scalar par_m_covid = r(c_2)
    scalar par_u_covid = r(c_3)

      centile par_sim_nonCovid, centile(2.5 50 97.5)
    scalar par_l_nonCovid = r(c_1)
      scalar par_m_nonCovid = r(c_2)
    scalar par_u_nonCovid = r(c_3)

      centile paf_total, centile(2.5 50 97.5)
    scalar paf_l_total = r(c_1)
      scalar paf_m_total = r(c_2)
    scalar paf_u_total = r(c_3)

    centile paf_covid, centile(2.5 50 97.5)
    scalar paf_l_covid = r(c_1)
      scalar paf_m_covid = r(c_2)
    scalar paf_u_covid = r(c_3)

      centile paf_nonCovid, centile(2.5 50 97.5)
    scalar paf_l_nonCovid = r(c_1)
      scalar paf_m_nonCovid = r(c_2)
    scalar paf_u_nonCovid = r(c_3)


    * 6. Store results back in main dataset
    restore

      replace par_total = par_m_total in `i'
    replace lower95_total = par_l_total in `i'
    replace upper95_total = par_u_total in `i'
```

```
    replace par_covid = par_m_covid in `i'
    replace lower95_covid = par_l_covid in `i'
    replace upper95_covid = par_u_covid in `i'

      replace par_nonCovid = par_m_nonCovid in `i'
    replace lower95_nonCovid = par_l_nonCovid in `i'
    replace upper95_nonCovid = par_u_nonCovid in `i'

      replace paf_total = paf_m_total in `i'
    replace paf_lower95_total = paf_l_total in `i'
    replace paf_upper95_total = paf_u_total in `i'

      replace paf_covid = paf_m_covid in `i'
    replace paf_lower95_covid = paf_l_covid in `i'
    replace paf_upper95_covid = paf_u_covid in `i'

      replace paf_nonCovid = paf_m_nonCovid in `i'
    replace paf_lower95_nonCovid = paf_l_nonCovid in `i'
    replace paf_upper95_nonCovid = paf_u_nonCovid in `i'

}
```

5 References

1. Kivimäki M, Lawlor DA, Smith GD, et al. Socioeconomic Position, Co-Occurrence of Behavior-Related Risk Factors, and Coronary Heart Disease: the Finnish Public Sector Study. *Am J Public Health* 2007; **97**(5): 874-9.
2. Korkeila K, Suominen S, Ahvenainen J, et al. Non-response and related factors in a nation-wide health survey. *Eur J Epidemiol* 2001; **17**(11): 991-9.
3. Sudlow C, Gallacher J, Allen N, et al. UK biobank: an open access resource for identifying the causes of a wide range of complex diseases of middle and old age. *PLoS Med* 2015; **12**(3): e1001779.
4. Kivimaki M, Nyberg ST, Batty GD, et al. Job strain as a risk factor for coronary heart disease: a collaborative meta-analysis of individual participant data. *Lancet* 2012; **380**(9852): 1491-7.
5. Kivimaki M, Strandberg T, Pentti J, et al. Body-mass index and risk of obesity-related complex multimorbidity: an observational multicohort study. *Lancet Diabetes Endocrinol* 2022; **10**(4): 253-63.
6. World Health Organization. WHO guidelines on physical activity and sedentary behaviour Geneva: World Health Organization; 2020. Licence: CC BY-NC-SA 3.0 IGO. 2020. Available at: https://www.who.int/publications/i/item/9789240015128 (accessed 30 January 2024.
7. Nyberg ST, Singh-Manoux A, Pentti J, et al. Association of Healthy Lifestyle With Years Lived Without Major Chronic Diseases. *JAMA Intern Med* 2020; **180**(5): 760-8.
8. Fransson EI, Heikkila K, Nyberg ST, et al. Job Strain as a Risk Factor for Leisure-Time Physical Inactivity: An Individual-Participant Meta-Analysis of Up to 170,000 Men and Women: The IPD-Work Consortium. *Am J Epidemiol* 2012; **176**(12): 1078-89.
9. Heikkila K, Fransson EI, Nyberg ST, et al. Job strain and health-related lifestyle: findings from an individual-participant meta-analysis of 118,000 working adults. *Am J Public Health* 2013; **103**(11): 2090-7.
10. Heikkilä K, Nyberg ST, Fransson EI, et al. Job Strain and Tobacco Smoking: An Individual-Participant Data Meta-Analysis of 166 130 Adults in 15 European Studies. *PLoS One* 2012; **7**(7): e35463.
11. UK Chief Medical Officers. UK Chief Medical Officers' low risk drinking guidelines 2016. https://www.gov.uk/government/publications/alcohol-consumption-advice-on-low-risk-drinking (accessed Feb 12 2020.
12. Nyberg ST, Batty GD, Pentti J, et al. Association of alcohol use with years lived without major chronic diseases: A multicohort study from the IPD-Work consortium and UK Biobank. *Lancet Reg Health Eur* 2022; **19**: 100417.
13. Goldberg DP, Gater R, Sartorius N, et al. The validity of two versions of the GHQ in the WHO study of mental illness in general health care. *Psychol Med* 1997; **27**(1): 191-7.
14. Virtanen M, Lallukka T, Ervasti J, et al. The joint contribution of cardiovascular disease and socioeconomic status to disability retirement: A register linkage study. *Int J Cardiol* 2017; **230**: 222-7.
15. Holi MM, Marttunen M, Aalberg V. Comparison of the GHQ-36, the GHQ-12 and the SCL-90 as psychiatric screening instruments in the Finnish population. *Nord J Psychiatry* 2003; **57**(3): 233-8.
16. Beck AT, Ward CH, Mendelson M, Mock J, Erbaugh J. An inventory for measuring depression. *Arch Gen Psychiatry* 1961; **4**: 561-71.

17. Nabi H, Kivimaki M, Suominen S, Koskenvuo M, Singh-Manoux A, Vahtera J. Does depression predict coronary heart disease and cerebrovascular disease equally well? The Health and Social Support Prospective Cohort Study. *Int J Epidemiol* 2010; **39**(4): 1016-24.
18. GBD 2017 Causes of Death Collaborators. Global, regional, and national age-sex-specific mortality for 282 causes of death in 195 countries and territories, 1980-2017: a systematic analysis for the Global Burden of Disease Study 2017. *Lancet* 2018; **392**(10159): 1736-88.
19. Nyberg ST, Batty GD, Pentti J, et al. Obesity and loss of disease-free years owing to major non-communicable diseases: a multicohort study. *Lancet Public Health* 2018; **3**(10): e490-e7.
20. Kivimaki M, Hamer M, Batty GD, et al. Antidepressant medication use, weight gain, and risk of type 2 diabetes: a population-based study. *Diabetes Care* 2010; **33**(12): 2611-6.
21. Nyberg ST, Fransson EI, Heikkila K, et al. Job strain as a risk factor for type 2 diabetes: a pooled analysis of 124,808 men and women. *Diabetes Care* 2014; **37**(8): 2268-75.
22. Fransson EI, Nyberg ST, Heikkila K, et al. Job strain and the risk of stroke: an individual-participant data meta-analysis. *Stroke* 2015; **46**(2): 557-9.
23. Heikkila K, Nyberg ST, Theorell T, et al. Work stress and risk of cancer: meta-analysis of 5700 incident cancer events in 116,000 European men and women. *Bmj* 2013; **346**: f165.
24. Heikkila K, Madsen IE, Nyberg ST, et al. Job strain and COPD exacerbations: an individual-participant meta-analysis. *Eur Respir J* 2014; **44**(1): 247-51.
25. Heikkila K, Madsen IE, Nyberg ST, et al. Job strain and the risk of severe asthma exacerbations: a meta-analysis of individual-participant data from 100 000 European men and women. *Allergy* 2014; **69**(6): 775-83.
26. Lean ME, Han TS, Morrison CE. Waist circumference as a measure for indicating need for weight management. *BMJ* 1995; **311**: 158-61.
27. NICE. Obesity: identification, assessment and management - Clinical guideline [CG189]: National Institute for Health and Care Excellence, 2022.
28. Townsend P, Phillimore P, Beattie A. Health and Deprivation: Inequality and the North. London: Routledge; 1988.
29. Smith DJ, Nicholl BI, Cullen B, et al. Prevalence and characteristics of probable major depression and bipolar disorder within UK biobank: cross-sectional study of 172,751 participants. *PLoS One* 2013; **8**(11): e75362.
30. UK Biobank Resource 158772: Derivation of mental states. https://biobank.ndph.ox.ac.uk/showcase/refer.cgi?id=158772 (accessed 18 Dec 2023.
31. UK Biobank Data-Field 2050: Frequency of depressed mood in last 2 weeks. https://biobank.ndph.ox.ac.uk/showcase/field.cgi?id=2050 (accessed 18 December 2023.
32. van der Meulen M, Amaya JM, Dekkers OM, Meijer OC. Association between use of systemic and inhaled glucocorticoids and changes in brain volume and white matter microstructure: a cross-sectional study using data from the UK Biobank. *BMJ Open* 2022; **12**(8): e062446.
33. Standards of medical care in diabetes--2010. *Diabetes Care* 2010; **33 Suppl 1**(Suppl 1): S11-61.
34. Sipilä PN, Heikkilä N, Lindbohm JV, et al. Hospital-treated infectious diseases and the risk of dementia: a large, multicohort, observational study with a replication cohort. *Lancet Infect Dis* 2021; **21**(11): 1557-67.
35. Sipila PN, Lindbohm JV, Batty GD, et al. Severe Infection and Risk of Cardiovascular Disease: A Multicohort Study. *Circulation* 2023; **147**(21): 1582-93.
36. Collaborators GBDAB. Global, regional, and national prevalence of adult overweight and obesity, 1990-2021, with forecasts to 2050: a forecasting study for the Global Burden of Disease Study 2021. *Lancet* 2025; **405**(10481): 813-38.
37. Collaborators GBDD. Global age-sex-specific mortality, life expectancy, and population estimates in 204 countries and territories and 811 subnational locations, 1950-2021, and the impact

of the COVID-19 pandemic: a comprehensive demographic analysis for the Global Burden of Disease Study 2021. *Lancet* 2024; **403**(10440): 1989-2056.
38. van Bueren S. Flexible imputation of missing data, second edn. *Chapman and Hall/CRC* 2018: 29–62.
39. Fine JP, Gray RJ. A Proportional Hazards Model for the Subdistribution of a Competing Risk. *Journal of the American Statistical Association* 1999; **94**: 496-509.
40. Therneau TM. A Package for Survival Analysis in R. R package version 3.8-3. <https://CRAN.R-project.org/package=survival>. 2024.

6 STROBE checklist

STROBE Statement—Checklist of items that should be included in reports of ***cohort studies***

	Item No	Recommendation
**Title and abstract**	1	(*a*) Indicate the study's design with a commonly used term in the title or the abstract **p.1**
		(*b*) Provide in the abstract an informative and balanced summary of what was done and what was found **p. 4-5**
**Introduction**		
Background/rationale	2	Explain the scientific background and rationale for the investigation being reported **p. 6**
Objectives	3	State specific objectives, including any prespecified hypotheses **p. 6**
**Methods**		
Study design	4	Present key elements of study design early in the paper **p. 6-7**
Setting	5	Describe the setting, locations, and relevant dates, including periods of recruitment, exposure, follow-up, and data collection **p. 6-7, Appendix 2**
Participants	6	(*a*) Give the eligibility criteria, and the sources and methods of selection of participants. Describe methods of follow-up **p 6-7, Appendix 2, Figure S1**
		(*b*) For matched studies, give matching criteria and number of exposed and unexposed **-**
Variables	7	Clearly define all outcomes, exposures, predictors, potential confounders, and effect modifiers. Give diagnostic criteria, if applicable **pp 7-8, Appendix 1, Appendix 2**
Data sources/ measurement	8*	For each variable of interest, give sources of data and details of methods of assessment (measurement). Describe comparability of assessment methods if there is more than one group **pp 7-8, Appendix 2**
Bias	9	Describe any efforts to address potential sources of bias **pp 9-10, Appendix 2**
Study size	10	Explain how the study size was arrived at **p 11, Figure S1, Appendix 2**
Quantitative variables	11	Explain how quantitative variables were handled in the analyses. If applicable, describe which groupings were chosen and why **p. 7-8, Appendix 2**
Statistical methods	12	(*a*) Describe all statistical methods, including those used to control for confounding **pp 9-10, Appendix 2**
		(*b*) Describe any methods used to examine subgroups and interactions **pp 9-10, Appendix 2**
		(*c*) Explain how missing data were addressed **pp 9-10, Appendix 2**
		(*d*) If applicable, explain how loss to follow-up was addressed
		(*e*) Describe any sensitivity analyses **pp 9-10, Appendix 2**
**Results**		
Participants	13*	(a) Report numbers of individuals at each stage of study—eg numbers potentially eligible, examined for eligibility, confirmed eligible, included in the study, completing follow-up, and analysed **p. 11, Appendix 2, Appendix 3**
		(b) Give reasons for non-participation at each stage **p. 11, Appendix 2**
		(c) Consider use of a flow diagram **Figure S1**
Descriptive data	14*	(a) Give characteristics of study participants (eg demographic, clinical, social) and information on exposures and potential confounders **Table 1, p 11, Appendix 2, Appendix 3**
		(b) Indicate number of participants with missing data for each variable of interest **Table 1. Appendix 2**
		(c) Summarise follow-up time (eg, average and total amount) **p. 11, Appendix 2**
Outcome data	15*	Report numbers of outcome events or summary measures over time **p. 11, Appendix 2**
Main results	16	(*a*) Give unadjusted estimates and, if applicable, confounder-adjusted estimates and their precision (eg, 95% confidence interval). Make clear which confounders

		were adjusted for and why they were included **p. 11, Figure 1, Appendix 2, Appendix 3**
		(*b*) Report category boundaries when continuous variables were categorized **p. 7, Appendix 2**
		(*c*) If relevant, consider translating estimates of relative risk into absolute risk for a meaningful time period
Other analyses	17	Report other analyses done—eg analyses of subgroups and interactions, and sensitivity analyses **pp 11-13, Figures 2-5, Appendix 2, Appendix 3**
**Discussion**		
Key results	18	Summarise key results with reference to study objectives **p. 13**
Limitations	19	Discuss limitations of the study, taking into account sources of potential bias or imprecision. Discuss both direction and magnitude of any potential bias **p. 15**
Interpretation	20	Give a cautious overall interpretation of results considering objectives, limitations, multiplicity of analyses, results from similar studies, and other relevant evidence **p. 15**
Generalisability	21	Discuss the generalisability (external validity) of the study results **p. 15**
**Other information**		
Funding	22	Give the source of funding and the role of the funders for the present study and, if applicable, for the original study on which the present article is based **p. 10, 16-17**

*Give information separately for exposed and unexposed groups.

Note: An Explanation and Elaboration article discusses each checklist item and gives methodological background and published examples of transparent reporting. The STROBE checklist is best used in conjunction with this article (freely available on the Web sites of PLoS Medicine at http://www.plosmedicine.org/, Annals of Internal Medicine at http://www.annals.org/, and Epidemiology at http://www.epidem.com/). Information on the STROBE Initiative is available at http://www.strobe-statement.org.
